# Chromobox protein homolog 7 suppresses the stem-like phenotype of glioblastoma cells by regulating the myosin heavy chain 9-NF-κB signaling pathway

**DOI:** 10.1038/s41420-025-02362-7

**Published:** 2025-02-23

**Authors:** Kaixiang Ni, Yuankun Liu, Pinggang DI, Lu Wang, Hui Huang, R. M. Damian Holsinger, Karrie Mei-Yee Kiang, Jiantong Jiao

**Affiliations:** 1 The Affiliated Wuxi People’s Hospital of Nanjing Medical University, Wuxi People’s Hospital, Wuxi Medical Center, Nanjing Medical University, Nanjing, China; 2 Department of Neurosurgery, The Affiliated Wuxi People’s Hospital of Nanjing Medical University, Wuxi People’s Hospital, Wuxi Medical Center, Nanjing Medical University, 214023 Wuxi, Jiangsu China; 3 Department of Emergency, Wulian County People’s Hospital, 262300 Rizhao, Shandong China; 4 Department of Pathology, The Affiliated Wuxi People’s Hospital of Nanjing Medical University, Wuxi People’s Hospital, Nanjing Medical University, 214023 Wuxi, Jiangsu China; 5 Laboratory of Molecular Neuroscience and Dementia, School of Medical Sciences, Faculty of Medicine and Health, The University of Sydney, Camperdown, NSW 2050 Australia; 6 Neuroscience, School of Medical Sciences, Faculty of Medicine and Health, The University of Sydney, Sydney, NSW 2006 Australia; 7 Division of Neurosurgery, Department of Surgery, School of Clinical Medicine, LKS Faculty of Medicine, The University of Hong Kong, Hong Kong, China

**Keywords:** CNS cancer, Neural stem cells

## Abstract

Cancer stem cells (CSCs) are significant factors in the treatment resistance and recurrence of glioblastoma. Chromobox protein homolog 7 (CBX7) can inhibit the progression of various tumors, but its impact on the stem cell-like properties of glioblastoma cells remains unclear. Clinically, low levels of CBX7 are associated with poor prognosis and increased distant metastasis in glioblastoma patients, and this low expression is caused by methylation of the CBX7 promoter. Our current research indicates that CBX7 plays a key role in suppressing the stem-like phenotype of glioblastoma. In this study, through bioinformatics analysis, we found that CBX7 is the most significantly downregulated member of the CBX family in glioblastoma and is closely associated with the stem-like phenotype of glioblastoma cells. We show that CBX7 promotes the degradation of myosin heavy chain 9 (MYH9) protein through the ubiquitin-proteasome pathway via the polycomb repressive complex 1 (PRC1) and suppresses the stem-like phenotype of glioblastoma cells by inhibiting the nuclear factor kappa-B (NF-κB) signaling pathway. Furthermore, overexpression of MYH9 in glioblastoma cells reverses the inhibitory effects of CBX7 on migration, proliferation, invasion, and stemness of glioblastoma cells. In summary, CBX7 acts as a tumor suppressor by inhibiting the stem cell-like characteristics of glioblastoma. The CBX7-MYH9-NF-κB signaling axis may serve as a potential therapeutic target for glioblastoma.

## Introduction

Glioblastoma is a highly aggressive and lethal brain tumor with poor prognosis and limited treatment options. Central to the resilience and recurrence of glioblastoma are glioblastoma stem cells (GSCs), a subpopulation of cells capable of self-renewal, differentiation, and resistance to conventional therapies. Understanding the regulatory mechanisms governing GSC properties is essential for developing effective glioblastoma treatments.

The Chromobox (CBX) family of proteins are essential components of the Polycomb repressive complex 1 (PRC1) and play crucial roles in regulating stem cell identity and function [1, 2]. For instance, the absence of CBX1 leads to alterations in constitutive pericentric heterochromatin, reductions in histone 3 lysine 9 trimethylation, and decreased levels of heterochromatic 1γ, which in turn disrupt the structural integrity and function of nucleoli during the cell cycle [3]. CBX2 has been shown to enhance the sphere-forming ability of glioblastoma by regulating the Phosphatidylinositol 3-Kinase/Akt Pathway signaling pathway [4]. CBX3, on the other hand, promotes the differentiation of stem cells into smooth muscle cells through its interaction with diaphanous homolog 1/serum response factor [5]. CBX4 maintains the slow-cycling, undifferentiated state of human epidermal stem cells by inhibiting stem cell activation via SUMOylation [6]. Furthermore, CBX5 is known to regulate the stem cell-like phenotype of lung cancer cells and can serve as a prognostic marker for lung cancer, also playing a key role in the differentiation of endothelial progenitor cells into endothelial cells [7, 8]. The deletion of CBX6 leads to a significant reduction in the expression of several critical pluripotency genes, such as *Rex1*, *Klf4*, *NANOG*, and *ERR-β* [9], thereby triggering cellular differentiation. CBX7 not only induces the generation of pluripotent stem cells but also promotes the self-renewal of hematopoietic stem cells and progenitors through both canonical and non-canonical pathways [10]. Lastly, CBX8 contributes to the stem-like phenotype of hepatocellular carcinoma by regulating bone morphogenetic protein 4 activation of the Mitogen-Activated Protein Kinase pathway [11]. In summary, the CBX family plays pivotal roles in the biological regulation of tumor stem cells [6, 12].

In this context, we aim to investigate the relationship between CBX family proteins and GSCs. Among the CBX family members, CBX7 plays different roles in various types of cancer. In bladder cancer, CBX7 inhibits the Aldo-keto reductase family 1 member B10 and subsequently stimulates Extracellular Signal-Regulated Kinase signaling, thereby suppressing bladder cancer progression [13]. However, in gastric cancer, CBX7 has been found to suppress p16 and activate the AKT-NF-kB signaling pathway to promote the stemness phenotype of gastric cancer cells [14, 15]. In breast cancer, CBX7 inhibits cell growth and metastasis through blocking the binding of Twist1 to EphA2 promoter [16]. Despite literature reporting that CBX7 suppresses glioblastoma migration through the activation of the Hippo signaling pathway [17], the potential mechanisms by which it regulates GSCs in glioblastoma remain unclear. Our research focuses on elucidating the regulatory mechanisms of CBX7 in GSCs and further identifying and validating its direct target genes in glioblastoma.

In this study, we report the regulatory function and mechanism of CBX7 in glioblastoma cells stemness. Our findings indicate that CBX7 suppresses stem cell-like biological behaviors in glioblastoma by inhibiting the NF-κB signaling pathway. In vitro experiments demonstrated that CBX7 can inhibit glioblastoma cell stemness. In vivo experiments confirmed that CBX7 suppresses tumor formation. Furthermore, we identified MYH9 as a novel downstream target gene of CBX7. Our results show that MYH9 drives the NF-κB signaling pathway to promote glioblastoma progression. We also clarified that the hypermethylation of the CBX7 promoter is mediated by DNA methyltransferases (DNMT1, DNMT3A). Immunohistochemistry (IHC) analysis of clinical glioblastoma tissue samples revealed that CBX7 expression can serve as an independent prognostic factor for glioblastoma patients. Overall, our study highlights the critical role of CBX7 in regulating glioblastoma cell stemness and provides new insights into its potential as a therapeutic target and prognostic marker.

## Results

### Frequent downregulation of CBX7 predicts poor prognosis in glioblastoma patients

**Fig. 1:**
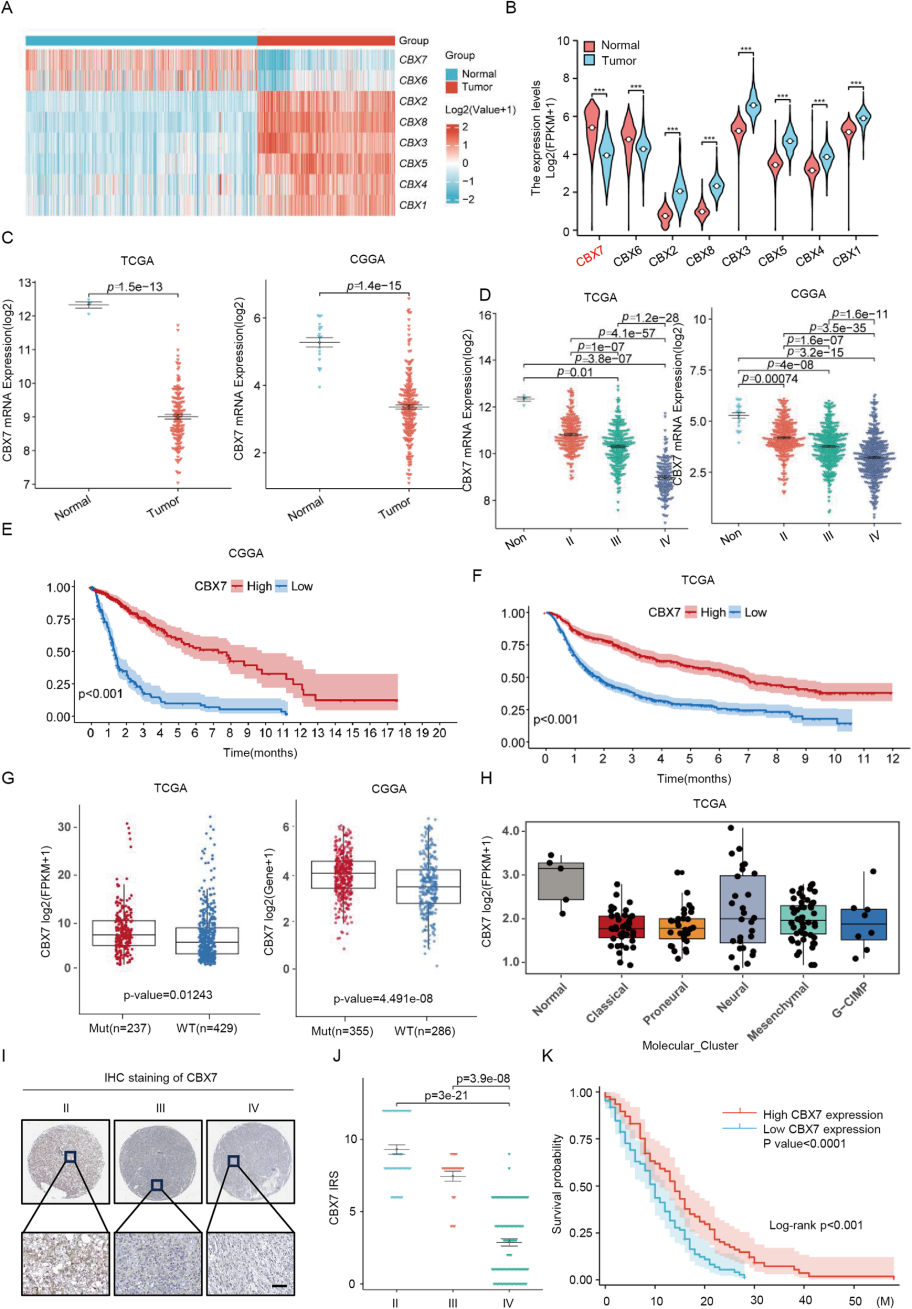
Frequent downregulation of CBX7 predicts poor prognosis in glioblastoma patients. **A** Clustering heat map of mRNA expression of the CBX family in TCGA-GTEx-GBM between tissues of Normal and Tumor. Heat map visualization performed using the ComplexHeatmap package. **B** Analysis of differences in mRNA expression of CBX family members in tumor and normal tissues. Data derived from the TCGA-GTEx-GBM dataset, and visualized using the ggplot2 package. Significant differences are observed across all members with increases in CBX1, 2, 3, 4, 5 and 8 and decreases in CBX6 and 7 (*n* = 1323, ***p < 0.001, Wilcoxon rank sum test). **C** The difference in mRNA expression of CBX7 in glioblastoma was analyzed using The Cancer Genome Atlas (TCGA) and the Chinese Glioma Genome Atlas (CGGA) databases. The data were obtained from the Gliovis online website (*p* values as indicated, Student’s *t* test). **D** Statistical analysis of CBX7 mRNA expression across different grades of gliomas derived from the datasets indicated in (**C**). (*p* values as indicated, two-tailed Student’s *t* test). **E**, **F** Overall survival analysis based on CBX7 mRNA expression in the indicated glioblastoma datasets (***p < 0.001, Kaplan–Meier survival test). **G** Box plot graphs show the differences in expression of CBX7 at the mRNA level of TCGA mutant glioma samples (Mut) and wild-type glioma samples (WT) (*p* values as indicated, Student’s *t* test). **H**. The box plot shows the expression of the CBX7 gene in different subtypes of glioblastoma in the TCGA database. **I** Immunohistochemical (IHC) analysis of CBX7 protein expression in a tissue array containing different grades of primary glioblastoma tissues. Scale bar = 20 μm. **J** CBX7 protein was frequently decreased in high grade glioblastoma compared with low grade glioblastoma (p values as indicated, Student’s *t* test). **K** Overall survival analysis based on CBX7 expression in glioblastoma tissues. Groups were ranked according to CBX7 IHC scores. The median expression of CBX7 was used as a cutoff (Log-rank *χ*^2^ = 5.823, ***p < 0.001).

To investigate the potential research value of different CBX family members in glioblastoma patients, we first analyzed the expression of CBX 1-8 in tumor and normal tissues using the GTEx dataset. Our analysis revealed that CBX7 was the most significantly downregulated member of the CBX family (Fig. 1A, B). We further examined CBX7 expression in primary tissues, downloading sequencing data of primary glioblastoma tissues from the CGGA and TCGA datasets via the Gliovis database (http://gliovis.bioinfo.cnio.es/). This analysis showed that CBX7 mRNA levels were significantly downregulated in glioblastoma compared to normal tissues (Fig. 1C). Further analysis of glioma datasets from the TCGA and CGGA datasets indicated that CBX7 mRNA levels were downregulated in both low-grade and high-grade gliomas (Fig. 1D). CBX7 mRNA levels were observed to be lowest in grade 4 gliomas (*p* < 0.01). Survival analysis predicted that patients with higher CBX7 expression had significantly longer overall survival (OS) compared to those with lower CBX7 expression (Fig. 1E, F; log-rank survival analysis). Additionally, based on pathological characteristics, glioblastoma is divided into four different subtypes: proneural, neural, classical, and mesenchymal, which indicate different survival rates for glioblastoma patients [18]. We examined the association between CBX7 expression and these glioblastoma subtypes and found that CBX7 expression varied among different subtypes and was significantly different from the normal group (Fig. 1H). Isocitrate dehydrogenase (IDH) mutations are a critical event in glioblastoma progression and is widely used as a diagnostic and prognostic marker for glioblastoma patients [19]. We explored the potential relationship between CBX7 and IDH mutation in glioblastoma in the TCGA and CGGA datasets, finding that CBX7 expression (*p* < 0.05) was positively correlated with IDH mutation, suggesting that CBX7 is a potential biomarker for predicting glioblastoma survival and IDH classification (Fig. 1G). These data collectively indicate that CBX7 is closely related to the pathological subtypes of glioblastoma and can be an important marker for glioblastoma classification (Fig. 1G, H). To further determine the clinical significance of CBX7 in human glioblastoma, we performed immunohistochemistry (IHC) on a glioblastoma tissue microarray containing 180 tumor samples. Representative images show that CBX7 was primarily localized in the cytoplasm, and quantitative analysis demonstrated that CBX7 expression varied among different grades of gliomas, with lower expression in higher-grade gliomas (Fig. 1I, J). Survival analysis based on median CBX7 levels showed that higher CBX7 levels were associated with better clinical outcomes, regardless of age and gender (Fig. 1K). In summary, our data suggest that CBX7 is frequently downregulated in glioblastoma and is significantly associated with poor prognosis in glioblastoma patients.

### CBX7 inhibits stemness in glioblastoma cells

**Fig. 2:**
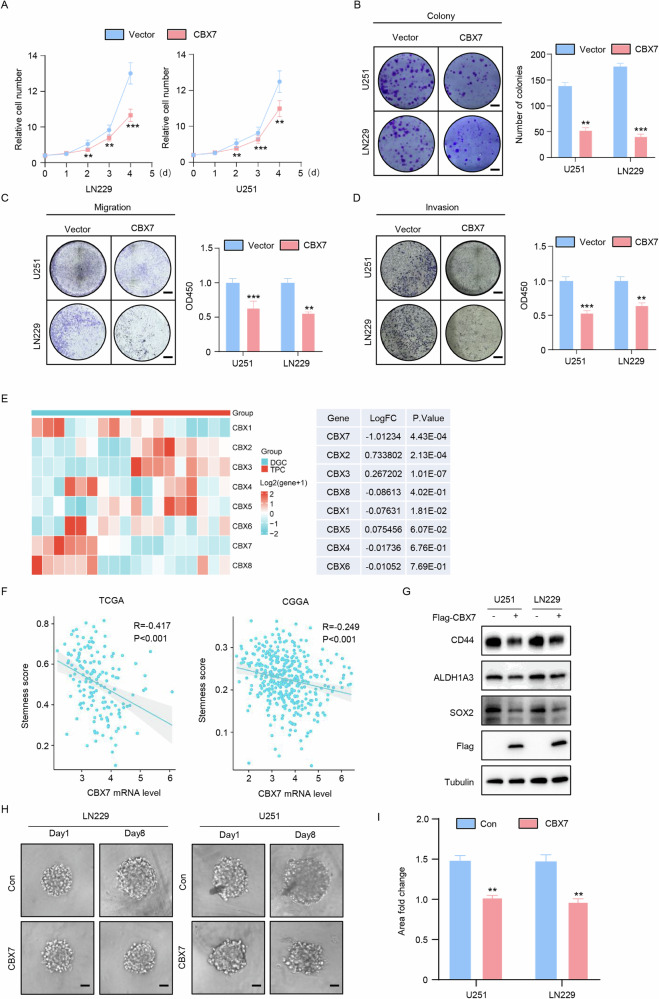
CBX7 inhibited glioblastoma cell stemness. **A** Cell growth assay of control (Vector) and CBX7 transfected LN229 and U251 cells using the Cell Counting Kit-8 demonstrated that the expression of CBX7 significantly decreases cell viability at all time points measured (mean ± SD, *n* = 4, **p < 0.01, ***p < 0.001, Student’s *t* test). **B** Colony formation (left) of control cells or those ectopically expressing CBX7 and the accompanying statistical analysis (right) (*n* = 4, **p < 0.01, ***p < 0.001, Student’s *t* test). **C**, **D**. Transwell invasion and migration assay of the indicated cells (left) and statistical analysis (right; *n* = 4, **p < 0.01, ***p < 0.001, Student’s *t* test,) demonstrate that transfection with CBX7 results in decreased levels of invasion and migration. **E** Clustering heat map of the expression of CBX family members at the mRNA level in non-stem glioma cells (DGC) and tumor propagating cells (TPC). Differentially expressed genes were calculated using the limma R package (p-values as indicated, Student’s t-test). Results demonstrate that CBX7 was the most significantly downregulated gene amongst the CBX family. **F** Correlation analysis between CBX7 mRNA levels and cell stemness scores in the specified dataset reveal a significant negative correlation between CBX7 mRNA expression and glioblastoma (*p*-values as indicated, Student’s *t* test). **G** Western blot analysis of CD44, ALDH1A3 and SOX2 in LN229 and U251 demonstrate a downregulation of these proteins following ectopic expression of CBX7. Tubulin served as a loading control. **H**, **I**. 3D-tumor spheroid growth was recorded (left) and quantitatively analyzed (right; *n* = 8, **p < 0.01, Student’s *t* test) and demonstrated that CBX7 expression resulted in significantly reduced sphere-forming capability in both U251 and LN229 cells. Scale bars = 200 μm.

To clarify whether CBX7 exerts tumor-suppressive biological functions in glioblastoma, we established CBX7 overexpression and knockdown models based on CBX7 protein levels in glioblastoma cell lines for subsequent studies (Fig. S1A, Supporting Information). Using RNAi technology and corresponding controls, we constructed knockdown (KD) models in A172 and T98G cells with relatively high endogenous CBX7 expression, while CBX7 overexpression (OE) models were generated in U251 and LN229 cell lines, which have lower CBX7 expression (Fig. S1B, Supporting Information), and verified by PCR (Fig. S1C, Supporting Information). Cell growth experiments demonstrated that CBX7 overexpression significantly inhibited the growth of glioblastoma cell lines (Fig. 2A). Subsequent functional experiments showed that CBX7 overexpression reduced clonogenicity and significantly decreased the migration and invasion potential of glioblastoma cells (Fig. 2B–D). Conversely, CBX7 knockdown promoted cell proliferation, colony formation, and significantly enhanced the migration and invasion potential of glioblastoma cells (Fig. S1D–G, Supporting Information). Since the CBX family plays a crucial role in regulating tumor cell stemness, we hypothesized that CBX7 might exert its biological function by regulating the stem cell phenotype in glioblastoma. To investigate this, we first selected a stemness dataset (GSE54791) related to glioblastoma patients from the GEO database to examine the correlation between the CBX family and glioblastoma stem cells. We found that compared to differentiated glioblastoma cells (DGC), expression of *CBX2*, *CBX3*, and *CBX5* were upregulated, while *CBX1*, *CBX4*, *CBX6*, *CBX7*, and *CBX8* were downregulated. Among them, *CBX7* was the most significantly downregulated gene in tumor-propagating cells (TPC) (Fig. 2E). Next, we analyzed the correlation between stemness genes and *CBX7* expression in glioblastoma using online clinical data. Based on gene set enrichment analysis (GSEA) of the combined CGGA-GBM and TCGA-GBM datasets, the ssGSEA enrichment score of HALLMARK_STEMNESS (stemness score) was significantly negatively correlated with CBX7 mRNA expression in glioblastoma (Fig. 2F). We first verified that the selected glioblastoma cell lines all had the potential for spheroidal stemness (Fig. S1G, Supporting Information). We also performed a 3D tumor sphere assay and observed the results at the same time points. The results showed that sphere-forming ability was reduced in CBX7-overexpressing U251 and LN229 cells, whereas it was significantly enhanced in CBX7-knockdown T98G and A172 cell lines (Fig. 2H, I, Fig. S1I, Supporting Information). Western blot results further demonstrated that the expression of stem cell markers (CD44, SOX2, ALDH1A3) decreased with CBX7 overexpression and increased with CBX7 knockdown (Fig. 2G, Fig. S2J, Supporting Information). In conclusion, these data suggest that CBX7 can inhibit stemness in glioblastoma cells.

### CBX7 promotes ubiquitin-dependent degradation of MYH9

**Fig. 3:**
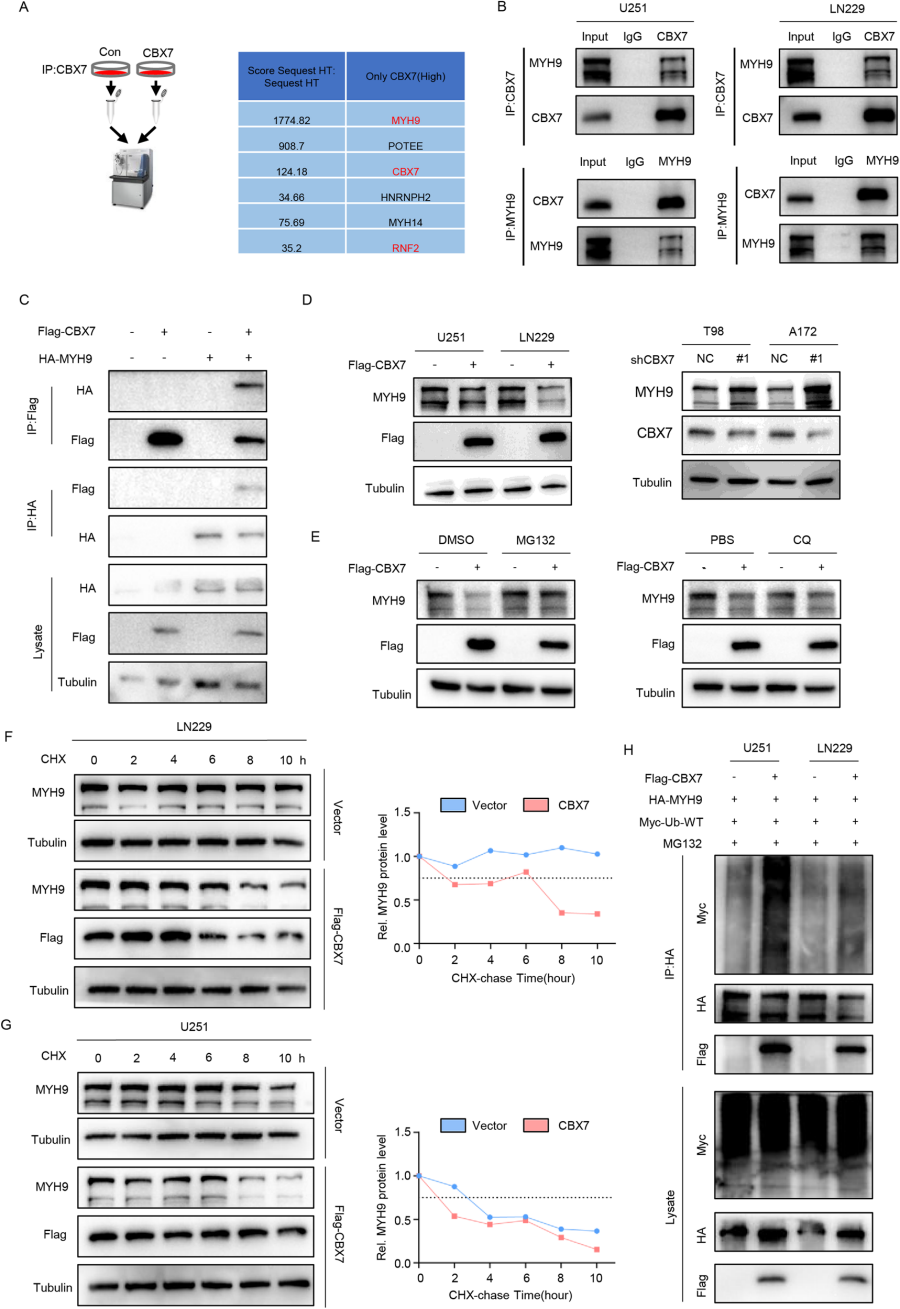
CBX7 promotes MYH9 degradation in the form of ubiquitination modification. **A** LC-MS/MS (Left) with an anti-FLAG-tag antibody and IgG in designated cells identified MYH9 as a potential binding target, as it had the highest peptide match score (sorted by Score Sequest HT(Right)) with CBX7. **B** Co-immunoprecipitation (Co-IP) followed by western blotting using anti-CBX7 or anti-MYH9 antibodies confirmed the endogenous association of CBX7 and MYH9 in U251 and LN229 cells. **C** Co-IP followed by western blotting using anti-Flag or anti-HA antibodies revealed the exogenous association of CBX7 and MYH9 in U251 and LN229 cells. **D** Expression of Flag-tagged CBX7 in either U251 or LN229 cells resulted in the downregulation of endogenous MYH9 levels. Knockdown of endogenous CBX7 using the shCBX7 silencing vector (#1) resulted in an upregulation of endogenous CBX7. NC refers to a scrambled silencing sequence that was used as a transfection control. Tubulin was used as a loading control. **E** Transfection of 293T cells with CBX7-overexpressing vector resulted in decreased expression of endogenous MYH9 and this was reversed following treatment with the proteasome inhibitor MG132 (left panel) whilst treatment with the lysosome inhibitor chloroquine (CQ) had no effect on MYH9 levels in the cells (right panel). **F**, **G** Treatment of CBX7-overexpressing LN229 (**F**) and U251 (**G**) cells with cycloheximide (CHX), used to block new protein synthesis, revealed that CBX7 overexpression significantly promoted the degradation of endogenous MYH9 protein. The relative levels of MYH9 protein at the indicated timepoints are shown in the panel on the right. **H** Co-transfection of U251 and LN229 cells with indicated plasmids revealed that CBX7 overexpression substantially increased the polyubiquitination levels of endogenous MYH9. Cell lysates were subjected to denaturing-IP, and the ubiquitination of MYH9 and the indicated proteins were detected by western blotting.

The downstream target genes of CBX7 in glioblastoma remain undefined. To address this, we transiently transfected U251 cells with a Flag-tagged CBX7 overexpressing adenoviral vector. Using co-immunoprecipitation (co-IP) with an anti-Flag antibody or IgG as a control, followed by liquid chromatography-tandem mass spectrometry (LC-MS/MS), we identified MYH9 as a potential target, as it had the highest peptide match score with CBX7 (Fig. 3A, Supplementary Table S1). To validate the mass spectrometry results, we performed co-IP in U251 and LN229 cells, confirming the endogenous interaction between CBX7 and MYH9 (Fig. 3B). Additionally, co-transfection of CBX7 and MYH9 in 293 T cells demonstrated the exogenous interaction between CBX7 and MYH9 (Fig. 3C). Western blot analysis revealed that CBX7 overexpression decreased MYH9 protein levels, whereas CBX7 knockdown increased MYH9 protein levels (Fig. 3D). However, analysis of the Gliovis database showed no significant correlation between CBX7 and MYH9 at the mRNA level (Fig. S2A, S2B, Supporting Information). RT-qPCR of transfected U251 and LN229 cells also indicated that CBX7 overexpression or knockdown did not affect MYH9 transcription levels (Fig. S2C, S2D, Supporting Information). We then treated U251 and LN229 cells with cycloheximide (CHX) to block protein synthesis and assessed MYH9 protein stability. The results showed that CBX7 overexpression significantly promoted the degradation of endogenous MYH9 protein (Fig. 3F, G). To determine whether CBX7-mediated MYH9 degradation occurs through the ubiquitin-proteasome or lysosome pathway, we transfected 293T cells with either a CBX7 overexpression plasmid or an empty vector, followed by treatment with the proteasome inhibitor MG132 or the lysosome inhibitor chloroquine (CQ). The results showed that MG132 reversed CBX7-mediated MYH9 degradation, while CQ did not, suggesting that CBX7 promotes MYH9 degradation via the ubiquitin-proteasome pathway (Fig. 3E). Additionally, co-transfection of CBX7, MYH9, and Ubiquitin (Ub) plasmids in U251 and LN229 cells revealed that CBX7 overexpression significantly increased the polyubiquitination levels of exogenous MYH9 (Fig. 3H). In conclusion, our results demonstrate that CBX7 promotes the polyubiquitination and degradation of MYH9 via the ubiquitin-proteasome pathway.

### CBX7 promotes ubiquitination and degradation of MYH9 in a PRC1-dependent manner

Degradation of proteins via the ubiquitin-proteasome system (UPS) generally involves two stages. The first stage is the interaction between the protein substrate, ubiquitin, and the ubiquitylating enzymes E1, E2, and E3. The second stage involves the degradation of the ubiquitin-modified protein substrate by the proteasome [20]. By co-transfecting 293T cells with CBX7, MYH9, K48-Ub, and K63-Ub plasmids, we found that CBX7 could promote K48-type ubiquitination of MYH9 and inhibit K63-type ubiquitination (Fig. S2E, Supporting Information). Given that CBX7 cannot function as an E3 ubiquitin ligase by itself, we hypothesized that CBX7 might promote MYH9 degradation by recruiting other E3 ubiquitin ligases. It is well-known that CBX7, as a key component of the classical PRC1 complex, can bind the E3 ubiquitin ligase RING1A/B to monoubiquitinate histone H2A. By screening and analyzing the MS data, we found that RING1A (RNF1) and RING1B (RNF2) were among the high-scoring differentially expressed genes (Fig. 3A). Therefore, we first considered that CBX7 could function in the form of the PRC1 complex, with RING1A/B acting as the E3 ligase. To determine whether RING1A/B performed the E3 function, we used the PRC1 inhibitor RB3 to verify whether it hindered the ubiquitination-promoting effect of CBX7 on MYH9. The results showed that the addition of the RB3 inhibitor reversed the ubiquitination-promoting effect of CBX7 on MYH9 (Fig. S2F, Supporting Information). These findings indicate that CBX7 promotes K48-type ubiquitination and inhibits K63-type ubiquitination of MYH9, and that ubiquitination and degradation of MYH9 are facilitated in a PRC1-dependent manner.

### CBX7 negatively regulates the NF-κB signaling pathway through MYH9

**Fig. 4:**
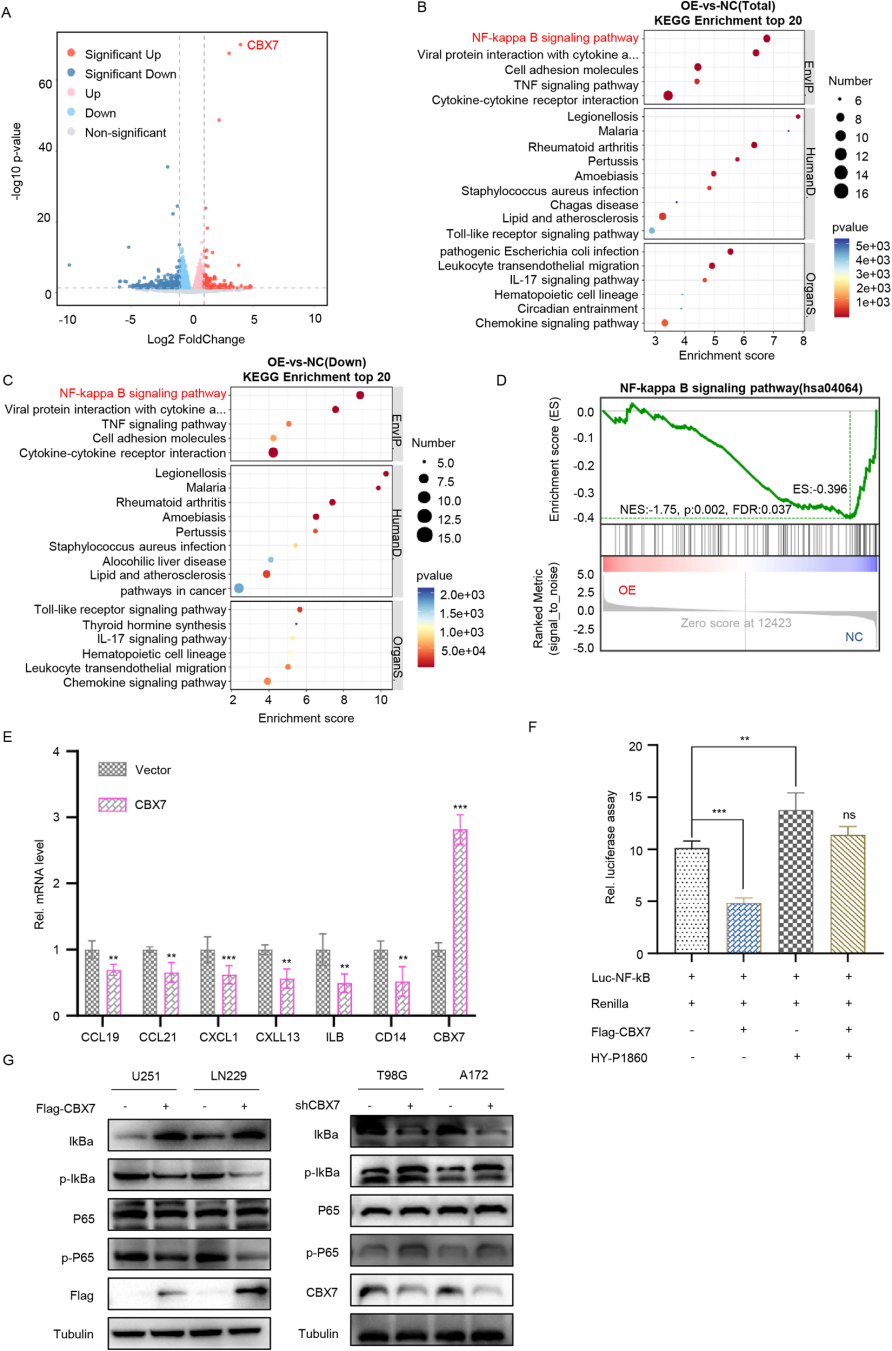
CBX7 regulates the NF-κB signaling pathway through MYH9. **A** Overexpression of CBX7 in the glioblastoma cell line U251 was confirmed via RNA sequencing (RNA-seq) 48 h following transfection. **B**, **C** KEGG pathway enrichment analysis indicated that the NF-κB signaling pathway was significantly enriched following CBX7 overexpression (**B**) and that overexpression of CBX7 resulted in notable suppression of the NF-κB signaling pathway (**C**). **D** Gene set enrichment analysis (GSEA) also revealed that overexpression of CBX7 in U251 cells leads to an inhibition of the NF-κB signaling pathway. **E** U251 cells were infected with control or AD-CBX7 for 36 h. Cells were collected for RT-qPCR analysis. (Data presented as mean ± SD with 3 replicates, **p < 0.01; ***p < 0.001, Student’s *t* test). **F** A luciferase assay to detect levels of NF-κB transcriptional activity in transformed U251 cells revealed that CBX7 overexpression results in an inhibition of NF-κB activity whilst the addition of HY-P1860 (20 ng/ml; *n* = 3), an NF-κB signaling pathway activator, added 24 h before the assay, led to a reversal of the inhibitory effects of CBX7 (**p < 0.01, ***p < 0.001, ns—not significant, Student’s *t* test). **G** Representative western blot analysis of NF-κB signaling pathway markers P65, IκBα and p-P65 in U251 and LN229 cells transfected with the indicated plasmids. Results demonstrate that CBX7 overexpression (Flag-CBX7) substantially inhibited the activation of NF-κB pathway markers (p65, IκBα), while CBX7 knockdown (shCBX7) increased their expression.

To further explore the downstream signaling pathways of the CBX7-MYH9 regulatory axis, we used blebbistatin, a specific inhibitor of MYH9 that suppresses the activation of the NF-κB signaling pathway [21]. To verify this result, we first co-transfected 293T cells with a luciferase reporter gene plasmid for NF-κB and an MYH9 overexpression plasmid. We found that MYH9 could increase the luciferase activity of NF-κB, but that the addition of blebbistatin inhibited the activating effect of MYH9 on NF-κB (Fig. S3A, Supporting Information). Subsequently, we confirmed through western blot that MYH9 overexpression activated the NF-κB signaling pathway markers (p65, IκBα), while MYH9 knockdown inhibited their activation (Fig. S3B, S3C, Supporting Information). These experiments demonstrated that MYH9 promotes the activation of the NF-κB signaling pathway. Next, we overexpressed CBX7 in the tumorigenic glioblastoma cell line U251 and performed RNA sequencing (RNA-seq) (Fig. 4A). KEGG pathway enrichment analysis revealed that the NF-κB signaling pathway was significantly enriched following CBX7 overexpression (Fig. 4B), and further analysis indicated that the NF-κB signaling pathway was notably suppressed (Fig. 4C). GSEA also showed that overexpression of CBX7 resulted in the suppression of the NF-κB signaling pathway (Fig. 4D). Subsequently, RT-qPCR confirmed that CBX7 overexpression inhibited the expression of downstream genes of the NF-κB pathway (*CCL19*, *CCL21*, *CXCL1*, *CXCL13*, *IL1B*, *CD14*) (Fig. 4E). Further validation showed that CBX7 overexpression could inhibit NF-κB activity as detected using a luciferase assay, and this inhibitory effect could be reversed by an NF-κB signaling pathway activator (Fig. 4F). Additionally, western blotting confirmed that CBX7 overexpression significantly inhibited the activation of NF-κB pathway markers (p65, IκBα), while CBX7 knockdown promoted their expression (Fig. 4G). Finally, to determine whether CBX7 inhibits the NF-κB signaling pathway by promoting the degradation of MYH9, co-transfection of MYH9 and CBX7 in U251 and LN229 cells revealed that MYH9 could reverse the inhibitory effect of CBX7 on the NF-κB signaling pathway. The dual luciferase reporter gene experiment also provided similar results (Fig. S3D, S3E, Supporting Information). Therefore, we conclude that CBX7 negatively regulates the NF-κB signaling pathway through MYH9.

### CBX7 inhibits the stemness of glioblastoma cells through MYH9

**Fig. 5:**
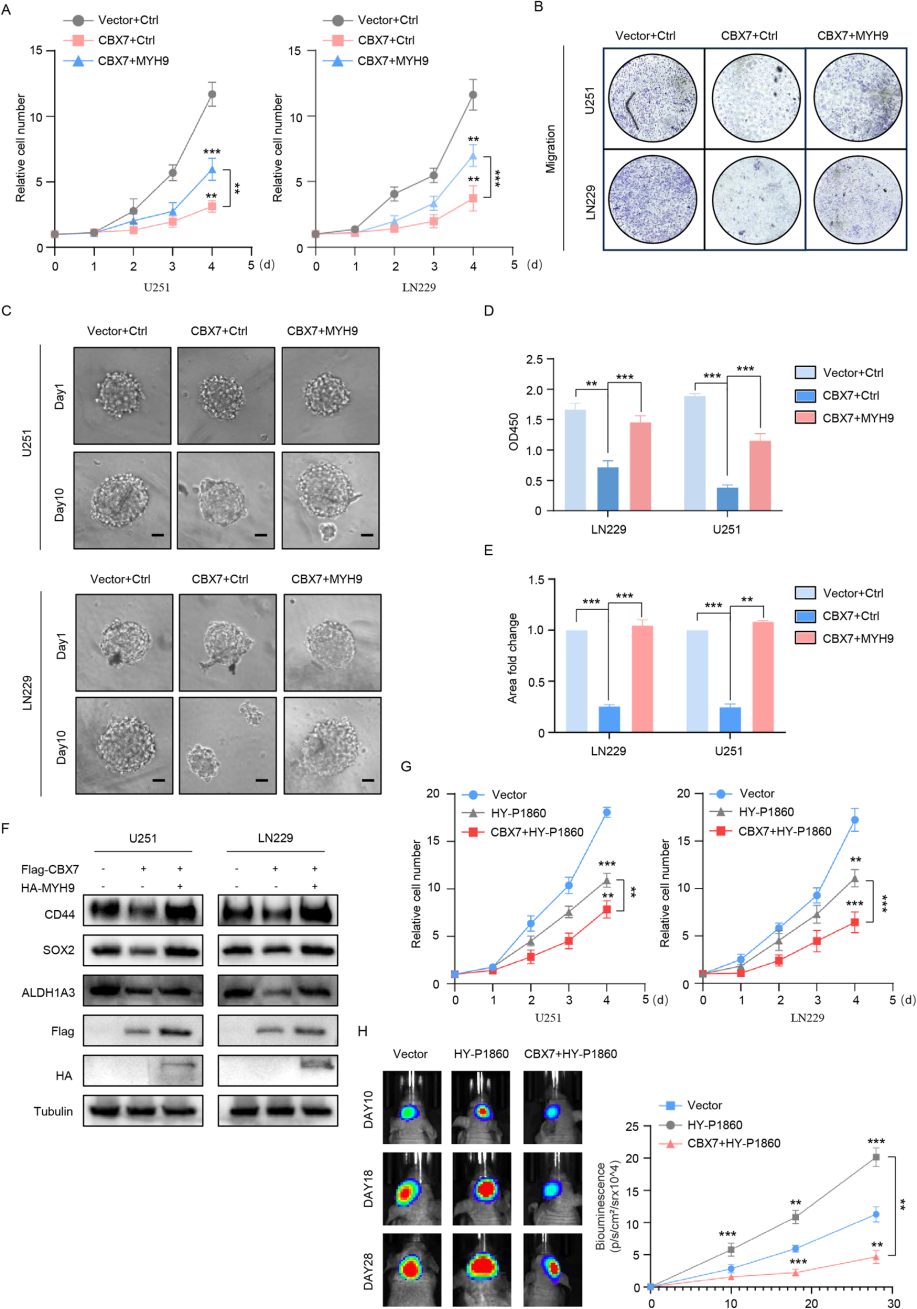
CBX7 inhibits glioblastoma cell stemness through MYH9. **A** Proliferation of glioblastoma cells transfected with the indicated plasmids was measured using CCK-8 (*n* = 4, **p < 0.01, ***p < 0.001, Student’s *t* test). Results reveal that transfection with a CBX7-overexpressing plasmid alone results in a significant reduction in stemness characteristics, whereas co-transfection with CBX7 and MYH9 partially reversed the inhibitory effects of CBX7. **B**, **D** Transwell migration assays (**B**) and the statistical analysis (**D**; *n* = 6, **p < 0.01, Student’s *t* test) reveal significant differences when CBX7 is co-transfected with MYH9 in both LN229 and U251 cells compared to when CBX7 is transfected by itself, which leads to significant decreases in stemness characteristics. **C**, **E** 3D-tumor spheroid growth was recorded (**C**) and quantitatively analyzed (**E**; *n* = 12, **p < 0.01, ***p < 0.001, Student’s *t* test) and illustrates the inhibitory effects of CBX7 compared to CBX7 co-transfected with MYH9 on spheroid growth. Scale bars, 200 μm. **F** Representative western blots of stem cell markers CD44, ALDHIA3, SOX2 in U251 and LN229 cells transfected with indicated plasmids. Transfection with CBX7 decreases expression of the stem cell markers whilst co-transfection of CBX7 and MYH9 stabilizes expression of the markers. **G** Measurement of proliferative capacity of CBX7 transfected cells revealed that whilst CBX7 significantly inhibited cell proliferation, treatment with an activator of the NF-κB signaling pathway, HY-P1860, significantly reversed the inhibitory effects. Proliferation was measured using the CCK-8 assay (*n* = 6, **p < 0.01, ***p < 0.001, Student’s *t* test). **H** To determine the in vivo effects of CBX7, we obtained bioluminescent images of intracranial xenografts derived from the implantation of the indicated cells (left) and statistical analysis of tumor volume (right; *n* = 9, Student’s *t* test, **p < 0.01, ***p < 0.001) revealed significant differences in the inhibitory effects of CBX7, indicating that CBX7 plays a biological role in tumor suppression by inhibiting activation of the NF-κB signaling pathway.

In 2021, Yao and colleagues reported that overexpression of Thrombospondin 1 (THBS1) promotes the proliferation, migration, and invasion of glioblastoma cells, while knockdown of MYH9 reverses these effects [22]. Additionally, a 2023 study reported that gambogic amide forms a complex with WD repeat domain 1, Cofilin, and MYH9, inhibiting the invasion of patient-derived glioma cells by disrupting the cytoskeleton. Overexpression of WD repeat domain 1 promotes the proliferation of patient-derived glioma cells, indicating a synergistic effect with MYH9 [23]. To investigate the biological functions of MYH9 in glioblastoma, we first discovered through a glioma chip (HBraG160Su01) that the expression of MYH9 increases with the pathological grade (Fig. S4A, S4B, Supporting Information). We then overexpressed MYH9 in U251 and LN229 cells and found that MYH9 could promote the proliferative and sphere-forming ability of glioblastoma cells (Fig. S4C, S4D, Supporting Information). Moreover, overexpression of MYH9 also promoted the increase of stemness markers (Fig. S4E, Supporting Information). These results indicate that MYH9 also promoted the malignant phenotype of glioblastoma cells. To explore whether CBX7 inhibits the stemness of glioblastoma cells by promoting the degradation of MYH9, we co-transfected glioblastoma cell lines with CBX7 and MYH9 overexpression plasmids and found that whilst overexpression of CBX7 significantly inhibited the growth, invasion, and stemness characteristics of glioblastoma cells, these effects were reversed by the overexpression of MYH9 (Fig. 5A–E). Western blot results showed that MYH9 could restore the inhibition of stem cell markers (CD44, ALDH1A3, SOX2) caused by CBX7 expression (Fig. 5F). In addition, we also demonstrated that HY-P1860, an activator of NF-κB signaling pathway, can promote tumor cell proliferation and CBX7 can inhibit its pro-cancer effect through in vitro and in vivo experiments, indicating that CBX7 plays a biological role in tumor suppression by inhibiting activation of the NF-κB signaling pathway (Fig. 5G, H). In summary, these results suggest that CBX7 inhibits the stemness phenotype of glioblastoma cells by downregulating MYH9.

### CBX7 inhibits the growth of tumor cells in vivo

**Fig. 6:**
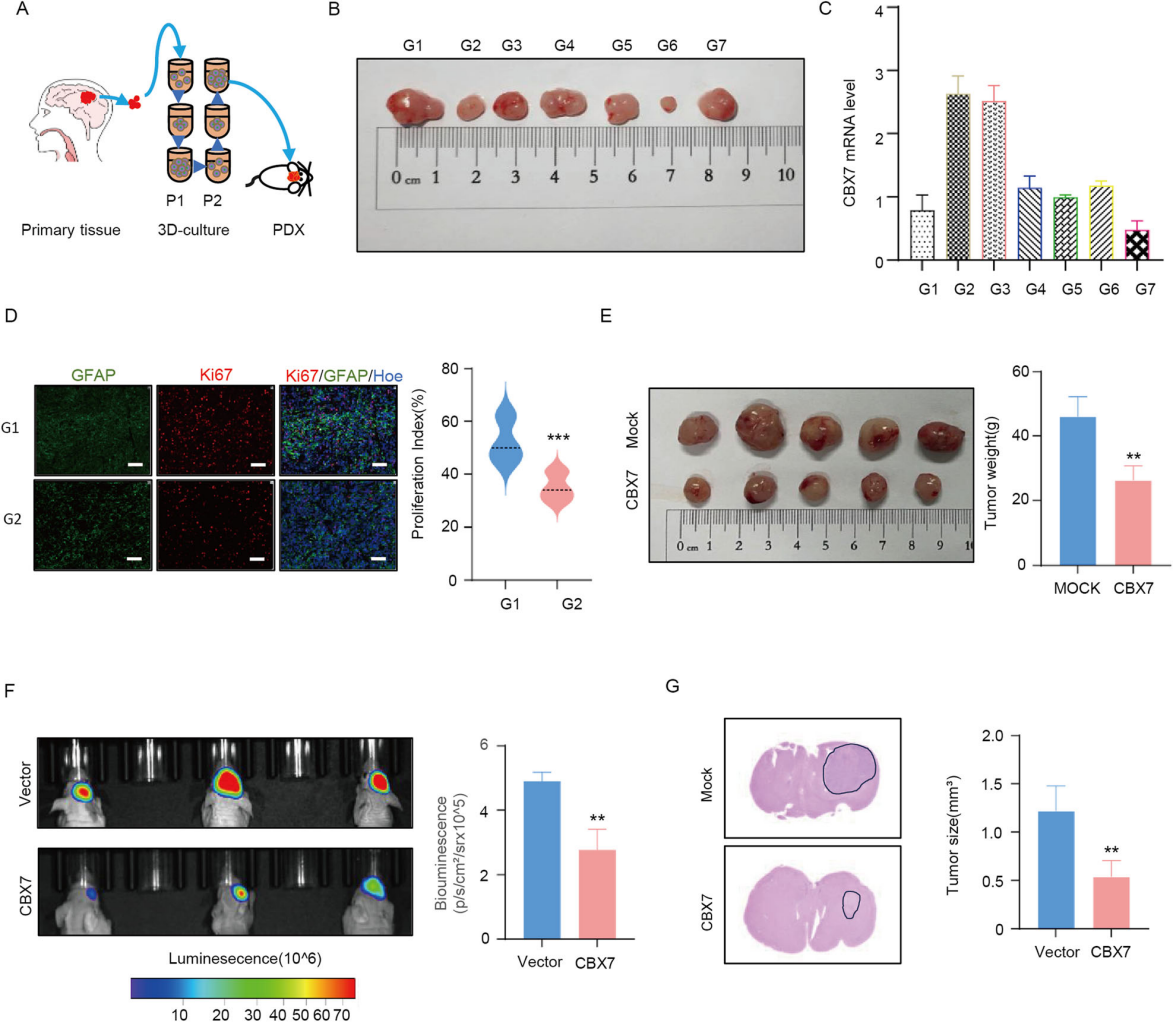
CBX7 inhibits the growth of tumor cells in vivo. **A** Illustration of the 3D-PDX model of patient derived glioblastoma tissues. The patient-derived glioblastoma cells were cultured in 3D and the second passages were used for xenograft models and subsequent analysis. **B**, **C**. Xenograft tumors derived from 3D-cultured patient-derived glioblastoma cells (**B**) and CBX7 mRNA expression levels in the xenografts (**C**). Analysis revealed that CBX7 mRNA expression levels in these cells were somewhat inversely correlated with tumor volume (except for G6), indicating that higher CBX7 levels are associated with reduced tumorigenicity. **D** Cell immunofluorescence analysis of Ki-67 expression (a marker of proliferation) in tumors derived from primary glioblastoma cell lines (left; at 20x magnification). Statistical analysis revealed that tumors formed by the glioblastoma primary cell line G1 exhibit a higher proliferative capacity (right; ***p < 0.001, Student’s *t* test). **E** Representative images of subcutaneous xenografts at day 21 following U251 injection. The right panel illustrates the tumor weight analysis, revealing that U251 cells overexpressing CBX7 resulted in a significant reduction in tumor volume and size (*n* = 5, **p < 0.01, Student’s *t* test). **F** Bioluminescent images of intracranial xenografts derived from the implantation of either vector or CBX7 expressing cells (left) and statistical analysis of tumor volume revealed that CBX7 overexpression significantly inhibited intracranial tumor development (right; *n* = 6, **p < 0.01, Student’s *t* test). **G** Representative hematoxylin and eosin (H&E) stained images demonstrating that overexpression of CBX7 reduces the volumes of xenograft tumors by more than 2-fold.

To determine the significance of CBX7 in human glioblastoma, we used a 3D culture method to derive primary cells from patient-derived glioblastoma tissue. Second-generation 3D cultures were used for xenograft models and subsequent analysis (Fig. 6A). To assess the correlation between CBX7 and glioblastoma tumorigenesis, we implanted second-generation 3D cultures subcutaneously into nude mice and measured CBX7 mRNA expression in the subcutaneous tumors by PCR. We found that CBX7 mRNA expression levels in these cells somewhat were inversely correlated with tumor volume; xenografts with higher CBX7 expression grew more slowly than those with lower CBX7 expression (except for G6), indicating that higher CBX7 levels are associated with reduced tumorigenicity (Fig. 6B, C). We further analyzed frozen sections of subcutaneous tumors derived from G1 and G2 cells, stained for proliferation markers, and found that tumors with high CBX7 expression exhibited lower proliferative capacity (Fig. 6D). Subsequently, we injected CBX7-overexpressing cells into the axillae of nude mice and observed a significant reduction in tumor volume and size in the CBX7-overexpressing group (Fig. 6E). To further validate the tumor-suppressive role of CBX7 in vivo, we performed intracranial xenograft tumor growth assays using luciferase-labeled U251 cells and control vectors in nude mice. The results showed that CBX7 overexpression significantly inhibited intracranial tumor development (Fig. 6F). Additionally, hematoxylin and eosin (H&E) staining of brain sections followed by volumetric measurements confirmed that the intracranial tumors were significantly smaller in the CBX7 overexpression group (Fig. 6G). In summary, these data support the tumor-suppressive function of CBX7 in vivo.

### High promoter methylation leads to frequent downregulation of CBX7 in glioblastoma

**Fig. 7:**
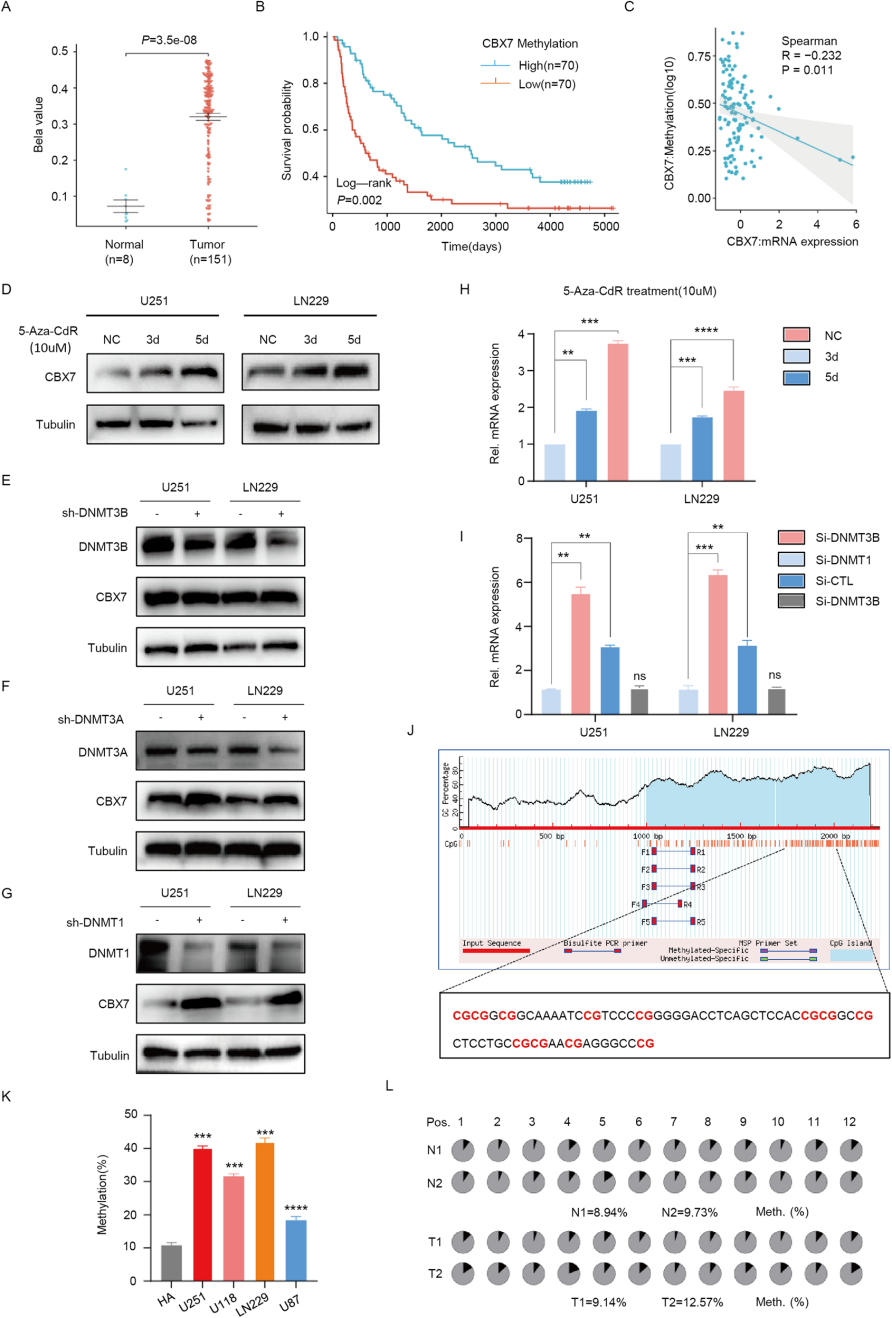
DNMT1 and DNMT3A mediate hypermethylation of the CBX7 promoter. **A** Beta values of *Cbx7* in control brain and Glioblastoma samples, from CGGA data set, plotted as a cellular map (*p* values are as indicated, Student’s *t* test). **B**. Log-rank survival curve based on the methylation levels of *CBX7* in the CGGA data set. The median expression of *CBX7* was used as a cutoff. Results show that patients with lower methylation levels had significantly better survival outcomes (Log-rank *χ*^2^ = 3.823, *p* = 0.002). **C** Correlation analysis, indicating a significant negative correlation between *Cbx7* methylation and its expression in the TCGA dataset (Spearman’s correlation analysis, *p* value and correlation coefficient R are as indicated). **D**, **H**. Protein (**D**) and mRNA levels (**H**) of CBX7 were restored in U251 and LN229 cells following treatment with 5-Aza-CdR at 10 μM for the indicated times (*n* = 6, **p < 0.01, ***p < 0.001, Student’s *t* test). **E**–**G** Western blotting analysis indicated that the knockdown of DNMT1 (**E**) and DNMT3A (**F**) in U251 and LN229 cells led to a significant increase in the level of CBX7. However, the knockdown of DNMT3B (**G**) did not have the same effect. **I** CBX7 mRNA levels were examined in DNMT1, DNMT3A, or DNMT3B-silenced U251 and LN229 cells by qRT-PCR (*n* = 8, **p < 0.01, ***p < 0.001, ns, not significant). Results revealed that whilst knockdown of DNMT1 and DNMT3A resulted in significant increases in the relative levels of CBX7 mRNA, knockdown of DNMT3B had no effect on CBX7 mRNA levels. **J** Design of primers targeting the promoter region of CBX7 for pyrosequencing. The solid box contains the primer sequences, and nucleotides in red indicate the 12 CG sites detected by the primers. **K**, **L** Methylation status of the CBX7 promoter in glioblastoma cell lines (**K**) and paired (**L**) glioblastoma tissues (T) and their adjacent noncancerous tissues (N). Percentage methylation of promoter was calculated as total percent of methylated cytosines from the 12 sites (*n* = 5, ***p < 0.001, Student’s *t* test). Pyrosequencing revealed that the methylation rate of CBX7 was higher in glioma cells (**K**) and in cancer tissues compared to adjacent non-cancerous tissues (**L**).

Studies have reported that the downregulation of CBX7 in glioblastoma is caused by hypermethylation of its promoter [17, 24], but the specific DNA methyltransferases (DNMTs) mediating CBX7 promoter hypermethylation have not been elucidated. To determine the cause of hypermethylation of the CBX7 promoter, we analyzed methylation data from the CGGA database and found that the methylation level of CBX7 was higher in tumor compared to normal tissues (Fig. 7A). Moreover, survival analysis showed that patients with lower methylation levels had better survival outcomes (Fig. 7B). Subsequently, a negative correlation between CBX7 mRNA and its methylation levels were observed in the TCGA database (Fig. 7C), indicating that promoter methylation plays a significant regulatory role in the expression of CBX7 in glioblastoma. Following this, we treated the U251 and LN229 cell lines with the DNA demethylating agent 5-Aza-CdR and observed an increase in CBX7 protein expression (Fig. 7D, H). Since members of the DNA methyltransferase (DNMT) family, including DNMT1, DNMT3A, and DNMT3B, play a crucial role in maintaining DNA methylation, we established knockdown models for these DNMTs in U251 and LN229 cells. Western blot analysis showed that the knockdown of DNMT1 and DNMT3A increased CBX7 protein levels, while DNMT3B did not have a similar effect (Fig. 7E–G), and PCR results also reached the same conclusion (Fig. 7I). These results indicate that DNMT1 and DNMT3A mediate the hypermethylation of the CBX7 promoter, leading to its frequent downregulation in glioblastoma. To further verify whether CBX7 is hypermethylated in glioma cell lines and tissues, we predicted the CpG islands in the CBX7 promoter region and designed corresponding primers (Fig. 7J). Pyrosequencing revealed that the methylation rate of CBX7 was higher in glioma cells (Fig. 7K), and clinical sample tests also showed that the methylation rate of CBX7 was higher in cancer tissues compared to adjacent non-cancerous tissues (Fig. 7L). In summary, we show that downregulation of CBX7 in glioblastoma is caused by promoter hypermethylation that is mediated by DNMT1 and DNMT3A.

## Discussion

CBX7 is an important component of PRC1 and is involved in the development of various cancers, including glioblastoma [17, 25]. In this study, query of the TCGA database revealed that CBX7 is the most significantly downregulated member of the CBX family in glioblastoma compared to normal tissues. Functional experiments showed that overexpression of CBX7 significantly inhibited glioblastoma cell growth, colony formation, migration, invasion, and stemness proliferation in vitro, and notably reduced the growth rate of intracranial and subcutaneous tumors in vivo. Mechanistically, CBX7 promotes the degradation of MYH9 protein in the form of the PRC1 complex, thereby inhibiting the NF-κB signaling pathway. The inhibitory effects of CBX7 on glioblastoma cell proliferation, migration, invasion, and stemness are reversed following MYH9 overexpression. Additionally, our study revealed that DNMT1 and DNMT3A mediate the hypermethylation of the CBX7 promoter, leading to its frequent silencing in glioblastoma cell lines and primary tumor tissues. Clinically, low levels of CBX7 expression are associated with poor prognosis in glioblastoma patients, increased self-renewal capacity, and enhanced proliferation of cancer cells. Our findings suggest that DNMT-mediated methylation is a promising target for glioblastoma treatment.

The stem cell phenotype refers to a series of characteristics exhibited by stem cells or cells with stem cell-like properties, which enable them to self-renew and differentiate into various cell types [26]. In glioblastoma, the stem cell phenotype is often considered to play a critical role in tumor initiation, drug resistance, and recurrence, making it an important focus of glioblastoma research [27]. In this study, based on the TCGA public database, we explored the differential expression of CBX family members in stemness gene sets and found that CBX7 was significantly downregulated in tumor-propagating cells (TPCs) compared to differentiated glioblastoma cells (DGCs), with the most pronounced downregulation among CBX family members. Subsequent in vitro functional experiments demonstrated that overexpression of CBX7 significantly inhibited tumor sphere growth, and western blot results also showed decreased expression of stemness markers. Our findings complement the understanding of the role of CBX7 in the stemness phenotype in glioblastoma.

We conducted mass spectrophotometry analysis of glioblastoma cells and identified MYH9 as a novel regulatory target of CBX7. While CBX proteins typically function as transcriptional repressors of downstream targets, analysis of sequencing data showed that MYH9 mRNA levels did not change with CBX7 overexpression, whereas CBX7 promoted MYH9 protein degradation. MYH9, also known as non-muscle myosin heavy chain IIA, belongs to the myosin II protein subfamily [28]. MYH9 participates in cell polarity, cell-cell adhesion, and cytoskeleton maintenance through its interaction with actin filaments and is involved in cancer invasion and metastasis [29]. MYH9 promotes tumor stemness by regulating various signaling pathways. For instance, MYH9 can ubiquitinate and degrade GSK3β, disrupting the β-catenin destruction complex and inducing stem-like phenotypes, epithelial-mesenchymal transition (EMT), and c-Jun signaling [30]. In non-small cell lung cancer, MYH9 activates the mTOR signaling pathway, enhancing stemness features [31]. MYH9 also serves as a marker and prognostic indicator for esophageal cancer stem cells, promoting tumorigenesis via the PI3K/AKT/mTOR axis [32]. Moreover, MYH9 plays a crucial role in maintaining and supporting hematopoietic stem cells and their progenitors [33]. Here, we reveal that CBX7 interacts with MYH9 to promote its degradation, thereby inhibiting glioblastoma cell stemness.

Recent research on MYH9 has focused on post-translational modifications [30, 34]. For example, in nasopharyngeal carcinoma, DNAJA4 directly interacts with MYH9, recruiting PSMD2 to promote MYH9 ubiquitination and degradation, thereby inhibiting nasopharyngeal carcinoma migration and EMT [35]. In triple-negative breast cancer, circRNA EIF6-224aa directly interacts with MYH9, inhibiting MYH9 degradation through the ubiquitin-proteasome pathway and activating the Wnt/β-catenin signaling pathway [36]. Conversely, lncRNA TPRG1-AS1 directly interacts with MYH9, promoting its degradation via the ubiquitin-proteasome pathway, thus hindering F-actin stress fiber formation and ultimately inhibiting human aortic smooth muscle cell migration [37]. In this study, we found that CBX7 interacts with MYH9, with Ring1A/B acting as E3 ligases, promoting MYH9 degradation through the ubiquitin-proteasome pathway in glioblastoma. Reportedly, Ring1A/B are two key subunits of the PRC1 complex, playing crucial roles in biological processes. They possess E3 ligase activity, catalyzing the monoubiquitination of histone H2A (H2AK119ub1), thus participating in chromatin structure regulation and repressing gene expression [38]. Ring1A/B also interact with Polycomb group (PcG) RING finger protein (PCGF) family members to form different PRC1 subcomplexes [38]. Here we reveal that CBX7, as part of the PRC1 complex, promotes MYH9 ubiquitination and degradation, thereby inhibiting the glioblastoma stem cell phenotype, with Ring1A/B serving as E3 ligases. However, further exploration is needed to determine the specific domains involved in the CBX7-MYH9 interaction and the specific sites of MYH9 ubiquitination by Ring1A/B.

To elucidate the downstream signaling pathways regulated by CBX7-MYH9, we prioritized the NF-κB signaling pathway in our transcriptome sequencing analysis. The NF-κB signaling pathway is a key regulatory network involved in immune responses, inflammation, cell proliferation, and differentiation in glioblastoma [39]. For instance, TRIM22 can activate the NF-κB signaling pathway by degrading IκBα, promoting glioblastoma cell proliferation [40]. Additionally, the NF-κB pathway can exacerbate the Warburg effect, leading to increased expression of LINC01127, activation of the MAPK4-JNK pathway, and promotion of glioblastoma stem cells [41]. Inhibition of the NF-κB pathway by curcumin significantly reduces glioblastoma cell proliferation and invasion and induces early G2/M cell cycle arrest [42]. In this study, we found that overexpression of CBX7 significantly inhibits, while MYH9 overexpression activates the NF-κB signaling pathway. The inhibitory effect of CBX7 on the NF-κB signaling pathway can be reversed by MYH9.

Overall, our findings reveal that DNA methyltransferases (DNMT1 and DNMT3A) mediate the hypermethylation of the CBX7 promoter, leading to its frequent downregulation in glioblastoma tissues and cell lines. The downregulation of CBX7 is associated with poor clinical outcomes. Additionally, we have demonstrated for the first time that CBX7, as part of the PRC1 complex, promotes MYH9 ubiquitination and degradation, inhibiting stemness, migration, invasion, and colony formation in glioblastoma cells. This newly identified CBX7-MYH9-NF-κB axis provides a novel therapeutic strategy for glioblastoma.

## Materials and methods

### Online cancer database analysis

We downloaded publicly available clinical and gene expression data from The Cancer Genome Atlas (TCGA) dataset (http://tcga-data.nci.ngov/tcga/) and the Chinese Glioma Genome Atlas (CGGA) database (http://www.cgga.org.cn). Pan-cancer data on *CBX7* gene expression were obtained from TCGA. In addition, stem and non-stem glioma data were obtained from GSE54791. Differentially expressed genes were calculated using the limma package in R. Survival analysis was performed using the survival package in R, and the cohort was divided into high expression and low expression groups based on the best Cut-off value.

### Clinical samples and immunohistochemical analysis

Immunohistochemical (IHC) staining was used to analyze the expression of CBX7 protein in glioblastoma tissues on a tissue microarray. Tissue microarrays were constructed by Shanghai Zhuoli Biotechnology Co., Ltd (ZL-BraG180sur01). The Ethics Committee of Shanghai Zhuoli Biotechnology Co., Ltd. Approved the study, and all subjects provided consent. Briefly, tissue slides were incubated with a rabbit anti-CBX7 antibody (1:5000; Abcam, Cambridge, UK), an anti-rabbit secondary antibody from Zymed Systems (1:10000; Invitrogen, Carlsbad, CA) and 3,3’-diaminobenzidine (DAB) to visualize the IHC labeling. The slides were lightly counterstained with cresyl violet. Normal rabbit IgG was used to confirm the specificity of the IHC. The expression of CBX7 was semi-quantitatively scored based on an established immunoreactivity scoring (IRS) system, with some modifications [43]. In summary, the IRS was calculated as the product of the proportion score (percentage of positive cells) and the staining intensity score (0-3). The proportion score represents the percentage of positively stained cells, whilst the intensity score represents the average intensity of the staining (0: no staining; 1: yellow; 2: claybank; and 3: tawny). Two pathologists blinded to the patient categories reviewed and scored the slides.

### Cell lines, primary cell preparation, and culture conditions

Human glioblastoma cell lines LN229, A172, and T98G were obtained from the American Type Culture Collection (ATCC). The U251 cell line and the human embryonic kidney cell line 293T were purchased from Pricell (Wuhan, China). All cells were cultured in Dulbecco’s Modified Eagle Medium (DMEM) supplemented with 10% Fetal Bovine Serum (FBS; CellBio, Australia). All cell lines were authenticated by short tandem repeat (STR) analysis and tested for mycoplasma contamination. For the patient-derived glioblastoma cell cultures, fresh glioblastoma tissue was collected immediately following tumor resection, washed, minced, and enzymatically dissociated. The tumor cells were resuspended and cultured in GBO medium to allow the formation of tumor spheres [44]. The primary spheres were cultured for 10 days to obtain a sufficient number of cells for passaging. Cells from the third or fourth generation were used for subsequent experiments, including tumor xenograft and western blot analysis.

### Vector construction and transduction

The full-length cDNA encoding human CBX7 and MYH9 were amplified by PCR and verified by DNA sequencing. The confirmed CBX7 cDNA was inserted into the lentiviral vector CMV-MCS-3FLAG-SV40-EGFP (Genechem, Shanghai, China). The MYH9 cDNA was inserted into the lentiviral vector with an HA tag, pCDH-CMV-MCS-EF1-PURO (Transheep, Shanghai, China). To achieve stable cell expression of CBX7, the indicated cells were transduced with the established lentivirus or the corresponding construct (Mock), and then selected with 2 μg/mL puromycin (Sangon Biotech, Shanghai, China). The short hairpin RNA (shRNA) targeting CBX7 and MYH9 were inserted into the vectors pGPU6/GFP/Neo-Homo-365 and pGPU6/GFP/Neo-Homo-259 respectively (GenePharm, Suzhou, Jiangsu, China).

### Immunoprecipitation assays

Immunoprecipitation (IP) experiments were conducted to analyze protein interactions. Briefly, cells were transfected with specified plasmids, and whole-cell lysates were precipitated using protein A/G microbeads (Santa Cruz, CA) with the specified antibodies. The precipitates were analyzed by western blotting using the indicated antibodies.

### Cell growth and colony formation assay

For the cell growth assay, a total of 1000 cells were seeded into a 96-well plate and monitored at specified time points using the Cell Counting Kit-8 (CCK-8) according to the manufacturer’s instructions (GlpBio, CA, USA). For the colony formation assay, 800 cells per well were seeded into six-well plates and maintained in complete medium for 14 days. Colonies were fixed with 4% paraformaldehyde (PFA) (Sangon, Shanghai, China), stained with 5% cresyl violet (Sangon, Shanghai, China), and the number of colonies was counted using an inverted microscope.

### EdU labeling assay

As previously described [45], DNA replication was analyzed using the 5-Ethynyl-2′-deoxyuridine (EdU) incorporation method. Briefly, cells were cultured in a 48-well plate for 24 h, incubated with EdU (Invitrogen) for 4 h, and then fixed. The staining procedure was performed according to the manufacturer’s instructions (Invitrogen, Carlsbad, CA). After staining, coverslips were mounted with Gelmount containing Hoechst 33342. The quantitative results are expressed as the percentage of EdU positive cells relative to the total number of cells/nuclei.

### Migration assay

A cell invasion assay was performed using Boyden Transwell chambers (8 mm pore size, BD Biosciences, Mountain View, CA) coated with Matrigel (50 μg, BD Biosciences) in a 48-well plate. Briefly, 1 × 10^5^ cells were seeded into the upper chamber, and medium containing 20% FBS was added to the lower chamber. After 36 h of incubation, the cells were fixed with 4% formaldehyde and stained with 5% cresyl violet. Cells on the upper side of the filter were carefully removed, and the cells on the underside of the filter were defined as invasive. Invasive cells were examined and counted using the EVOS cell imaging system (Life Technologies, Grand Island, NY, USA). Cell invasion was expressed as the percentage of migrating cells relative to the total area.

### 3D-tumor spheroid assay

According to the 3D spheroid formation and growth protocol, 1000 cells were seeded in a spheroid microplate (Corning Inc., NY, USA). Growth of the 3D spheroid-cultured cells was monitored using a live-cell imaging system (EVOS^®^ FL Auto Imaging System, Life Technologies, Carlsbad, CA, USA). For spheroid growth assessment, images of the tumor spheroids at designated time points were captured and spheroid diameter was measured to reflect spheroid growth.

### Reverse transcription PCR (RT-PCR) and Quantitative PCR (RT-qPCR)

Following manufacturer’s instructions, cellular RNA was extracted using Trizol^®^ reagent (at no. 15596026, Invitrogen, Carlsbad, CA, USA). cDNA was synthesized using the TaqMan Reverse Transcription Kit (Cat no. 4366596, Applied Biosystems) following the manufacturers protocol. PCR analysis for quantifying mRNA expression was performed on the LightCycler 480 II instrument (Roche Applied Science) using the Real SYBR Mixture (Cat no. 4309155, Invitrogen, Carlsbad). The endogenous control, glyceraldehyde-3-phosphate dehydrogenase (GAPDH), expression was monitored. Each experiment was conducted in triplicate. The relative mRNA expression was calculated using the 2^−ΔΔCt^ method. Specific oligonucleotide primer pairs are listed in Table S2, Supporting Information.

### mRNA sequencing and analysis process

The total transcriptome RNA-seq analysis was performed on the Illumina Novaseq 6000 sequencing platform at OE Biotech Co., Ltd. (Shanghai, China) to detect the mRNA expression profile of U251 cells overexpressing CBX7. Clean reads were aligned to the reference genome using HISAT2 software, generating raw counts corresponding to each known gene (in total 23899), and calculating gene expression levels (FPKM). Differential expression gene analysis was conducted using the DESeq2 software, where genes meeting the criteria of a *q*-value < 0.05 and fold change > 2 were defined as differentially expressed genes (DEGs). Kyoto Encyclopedia of Genes and Genomes Pathway enrichment analysis and GSEA enrichment analysis of the differentially expressed genes were performed based on the hypergeometric distribution algorithm and GSEA software.

### Pyrosequencing for CBX7 methylation levels

DNA methylation assays were performed by pyrosequencing of the *CBX7* gene. Briefly, genomic DNA was extracted from frozen patient glioma tissue and glioblastoma cell samples and bisulfite transformed. The bisulfite-converted DNA was then amplified and bound to a single-stranded template. Pyrosequencing runs were performed using the PyroMark Q96/48 ID System (QIAGEN, Hilden, Germany). Further details are provided elsewhere [46]. As mentioned above, methylation levels are expressed as the ratio of methylated cytosine to the sum of methylated and unmethylated cytosine at the 2-cytosine position. Primer sequences are provided in Table S3, Supporting Information.

### Luciferase reporter assay

We employed the NF-κB promoter luciferase reporter plasmid (MiaoLing; Wuhan, China) to evaluate the effect of CBX7 on NF-κB transcriptional activity. Briefly, the indicated cells were transiently transfected with the vector. The pRL-TKRenilla luciferase plasmid (Promega) was also transfected to serve as a control for the total amount of transfected DNA in each well in all experiments. Forty-eight hours post-transfection, the cells were harvested and luciferase activity was measured using the Dual-Luciferase^®^ Reporter Assay System (Promega).

### Western blot assay and chemical agents

Protein expression was determined using standard western blotting. Antibodies and chemical reagents used to detect the indicated proteins are listed in Table S4, Supporting Information.

### Tumor sphere formation and growth assay

Briefly, cells were seeded in 6-well plates (1000 cells/well) and maintained in NSC medium (DMEM/F-12 with 20 ng/ml bFGF (PeproTech, Rocky Hill, NJ), 20 ng/ml EGF (PeproTech), N2 (Thermo) and B27 (Thermo) to facilitate sphere formation. Images of the tumor spheres were obtained at designated time points, and sphere diameters were measured. At least 10 random fields were analyzed per experiment.

### Animal experiments

Four-week-old female nude mice were purchased from the Shanghai Institute of Biological Sciences, Chinese Academy of Sciences, and were housed under specific pathogen-free conditions. All mice were randomly assigned. For the U251 intracranial model, cells expressing the firefly luciferase gene were infected with either CBX7-overexpressing lentivirus or control lentivirus. A total of 5 × 10^5^ cells were stereotaxically implanted into the brains of individual mice. Tumor growth was monitored using bioluminescence imaging using the IVIS Spectrum system and quantified with Living Image Software. Additionally, to assess the tumor-suppressive effect of the CBX7 construct, a total of 5 × 10^6^ U251 cells expressing ectopic CBX7 or an empty vector were injected into the flanks of each nude mouse (n = 10), with tumor growth assessed by measuring tumor diameter every three days. At the end of the experiment, mice were euthanized by an overdose of anesthetic. The whole brains or tumors were extracted, paraffin-embedded, sectioned, and stained with hematoxylin and eosin (H&E). Images were captured using a laser scanning confocal microscope. All animal care and handling procedures were conducted in accordance with the National Institutes of Health’s Guide for the Care and Use of Laboratory Animals. All animal experiments were approved by the Ethics Committee of the Affiliated Wuxi People’s Hospital of Nanjing Medical University (No. (2024)235). Animal experiments were performed by two technicians who were blinded to the treatment conditions of the mice. Collection of human glioblastoma samples were approved by the Ethics Committee of the Affiliated Wuxi People’s Hospital of Nanjing Medical University (No. (2024)133).

### Statistical analysis

The experiments were independently repeated at least three times, and data are presented as mean ± standard deviation (SD). All data were analyzed using the SPSS 20.0 software package (IBM Corp., Boston, MA, USA) and were consistent with normal distribution and homogeneity of variance tests. The *t* test was used to compare differences between two groups, while one-way ANOVA was used for mean comparisons among multiple sample groups, followed by Tukey’s post hoc test for multiple comparisons within the groups. A P-value of less than 0.05 was considered statistically significant. Graphs were analyzed using GraphPad Prism 9.5 software (GraphPad Software, Boston, MA, USA).

## Supplementary information

Supplementary Figures and legends

Table S1

Table S2

Table S3

Table S4

Original western blots

## Data Availability

RNA-seq data generated for this study are available in the Gene Expression Omnibus Database (No. GSE283387). All data accessed from external sources and prior publications have been referenced in the text and corresponding figure legends. Additional data will be made available upon request. The data that support the findings of this study are available from the corresponding author upon reasonable request.
