# Supplementary material for: Chromobox protein homolog 7 suppresses the stem-like phenotype of glioblastoma cells by regulating the myosin heavy chain 9-NF-κB signaling pathway
Source: Cell Death Discov. 2025 Feb 23;11:74. doi: 10.1038/s41420-025-02362-7 (PMC11847914; doi:10.1038/s41420-025-02362-7)
Supplement: Supplementary file 1 — Supplementary Figures and legends [file 41420_2025_2362_MOESM1_ESM.pdf]

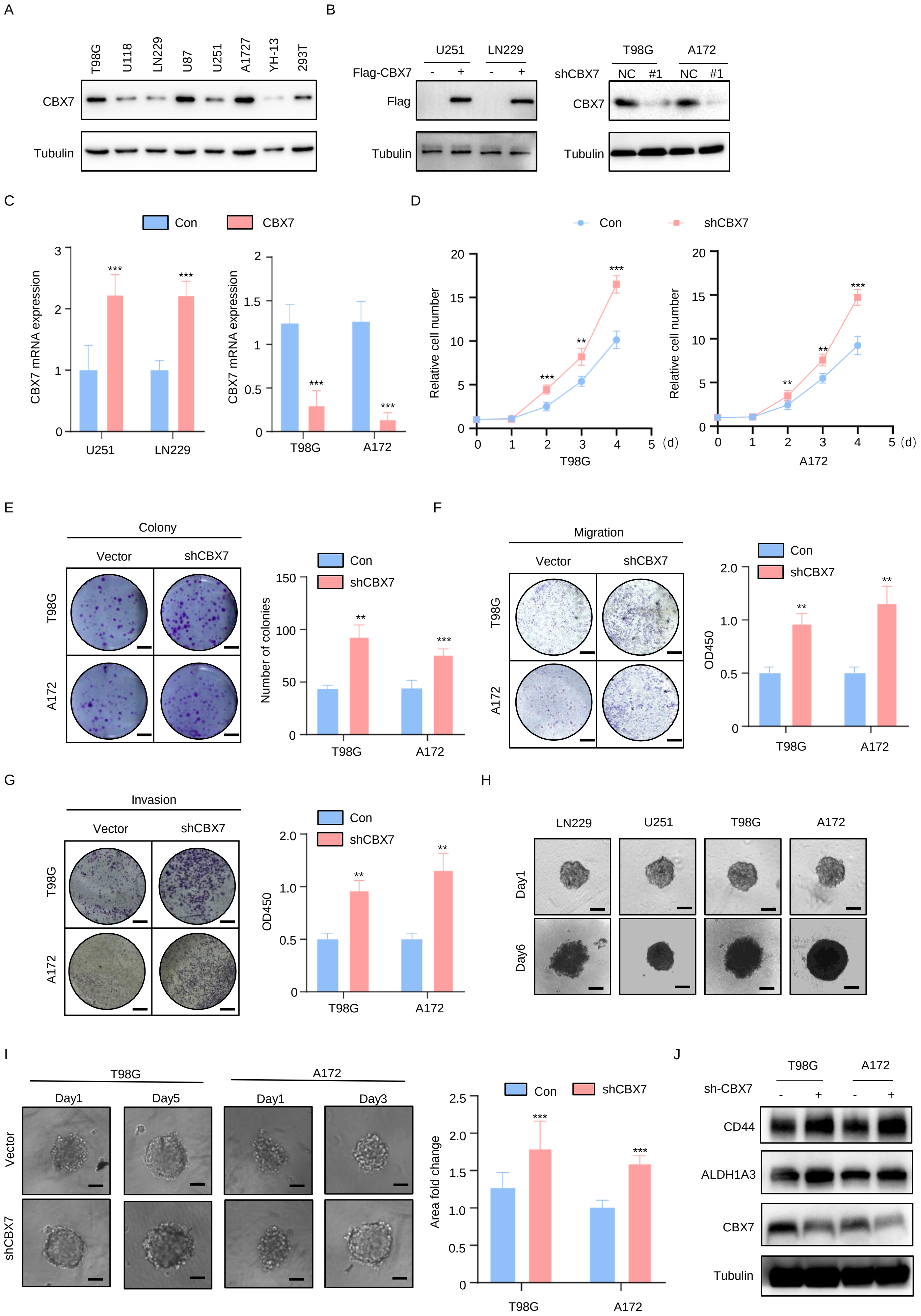
A
T98G
U118
LN229
U87
U251
A1727
YH-13
293T
CBX7
Tubulin
B
U251
LN229
Flag-CBX7
-
+
Flag
Tubulin
T98G
A172
shCBX7
NC
#1
CBX7
Tubulin
C
Con
CBX7
CBX7 mRNA expression
***
U251
LN229
T98G
A172
D
Con
shCBX7
Relative cell number
**
(d)
T98G
A172
E
Colony
Vector
shCBX7
Number of colonies
F
Migration
OD450
G
Invasion
H
LN229
U251
T98G
A172
Day1
Day6
I
Day1
Day5
Day3
Vector
shCBX7
Area fold change
J
sh-CBX7
CD44
ALDH1A3
CBX7
Tubulin

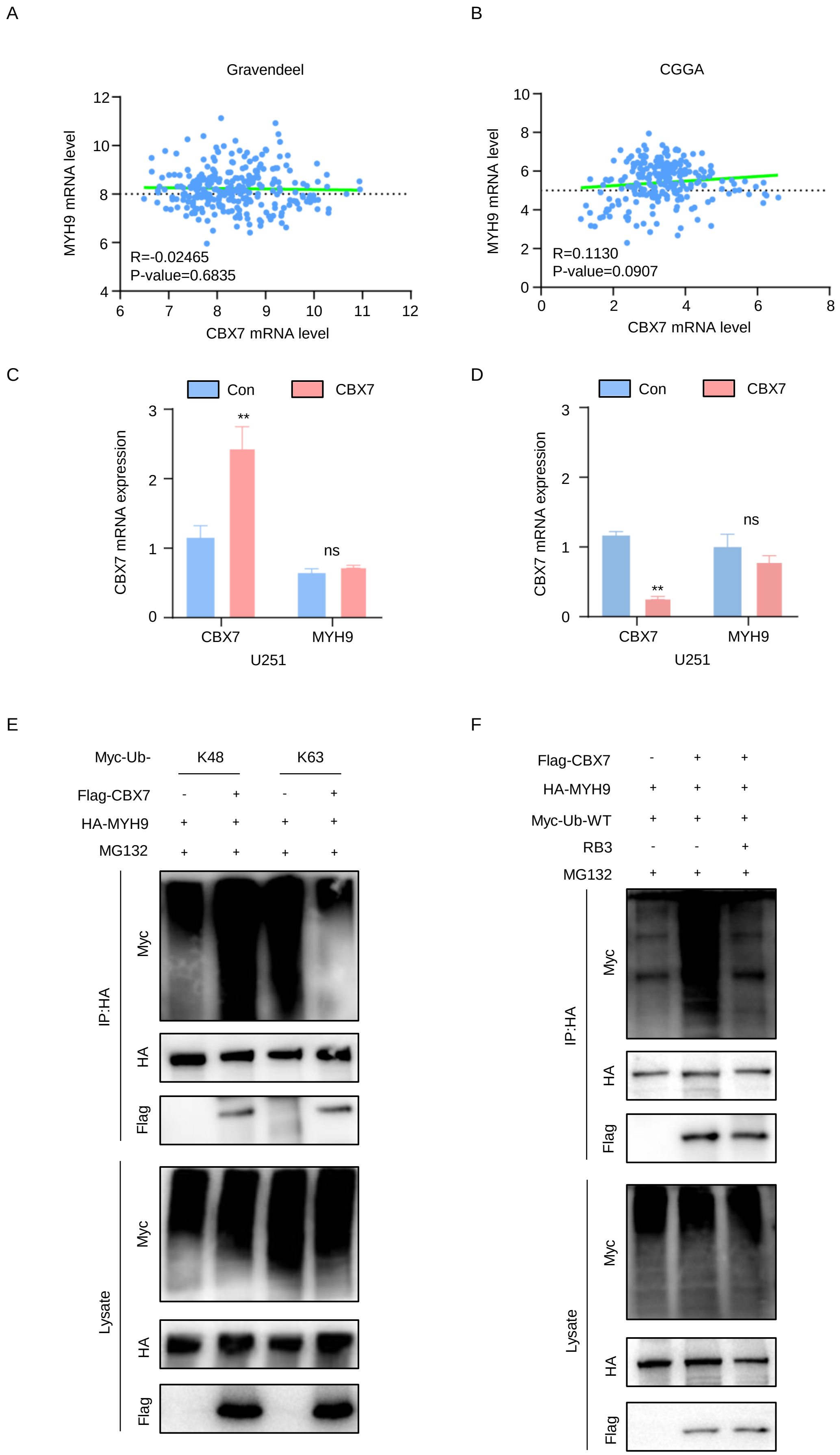
A
Gravendeel
MYH9 mRNA level
CBX7 mRNA level
R=-0.02465
P-value=0.6835
B
CGGA
MYH9 mRNA level
CBX7 mRNA level
R=0.1130
P-value=0.0907
C
Con
CBX7
CBX7 mRNA expression
**
ns
CBX7
MYH9
U251
D
Con
CBX7
CBX7 mRNA expression
**
ns
CBX7
MYH9
U251
E
Myc-Ub-
K48
K63
Flag-CBX7
HA-MYH9
MG132
IP:HA
Myc
HA
Flag
Lysate
Myc
HA
Flag
F
Flag-CBX7
HA-MYH9
Myc-Ub-WT
RB3
MG132
IP:HA
Myc
HA
Flag
Lysate
Myc
HA
Flag

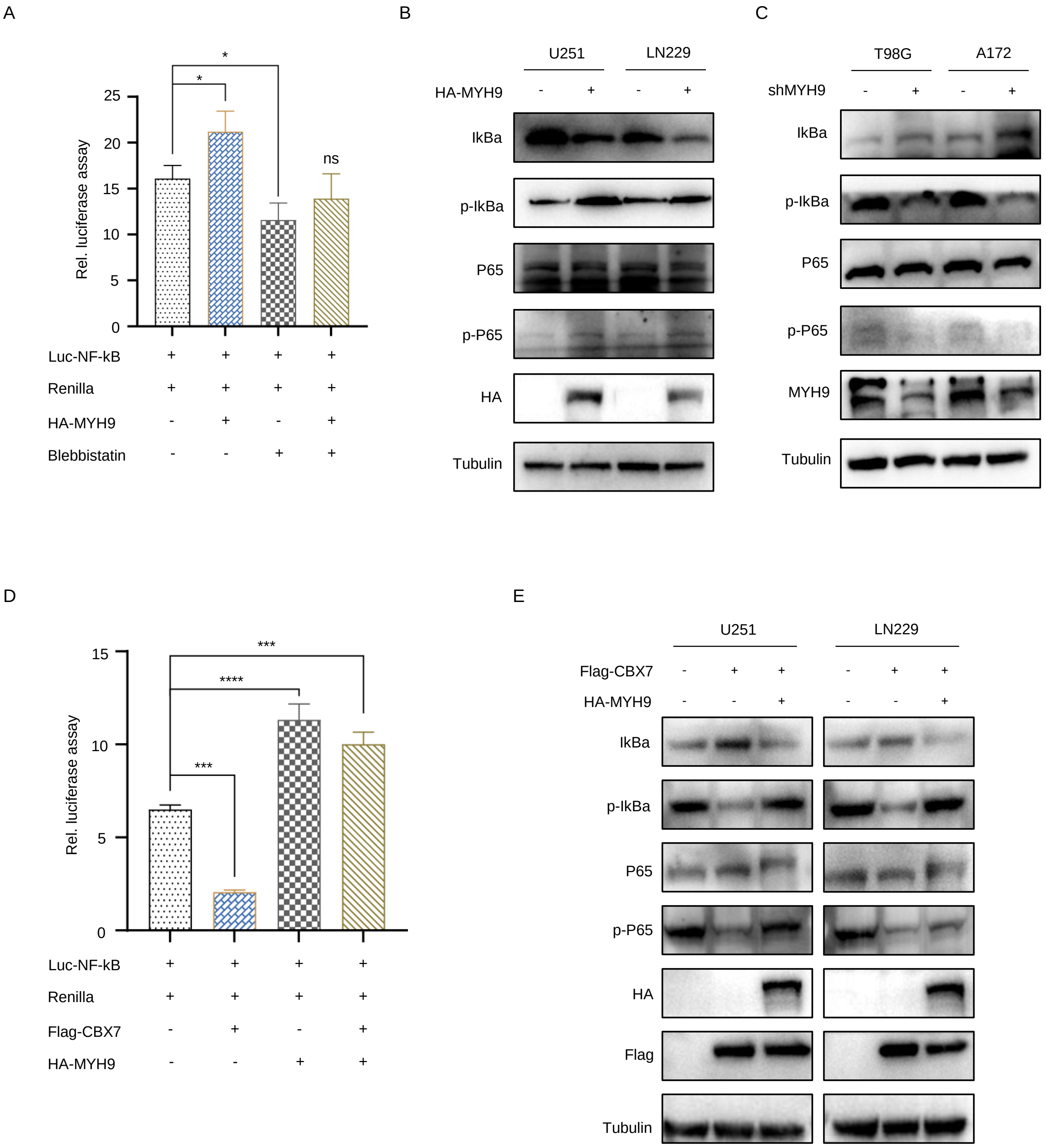
A
B
C
*
*
ns
25
20
15
10
5
0
Rel. luciferase assay
Luc-NF-kB + + + +
Renilla + + + +
HA-MYH9 - + - +
Blebbistatin - - + +
U251
LN229
HA-MYH9 - + - +
IkBa
p-IkBa
P65
p-P65
HA
Tubulin
T98G
A172
shMYH9 - + - +
IkBa
p-IkBa
P65
p-P65
MYH9
Tubulin
D
E
***
****
***
15
10
5
0
Rel. luciferase assay
Luc-NF-kB + + + +
Renilla + + + +
Flag-CBX7 - + - +
HA-MYH9 - - + +
U251
LN229
Flag-CBX7 - + + - + +
HA-MYH9 - - + - - +
IkBa
p-IkBa
P65
p-P65
HA
Flag
Tubulin

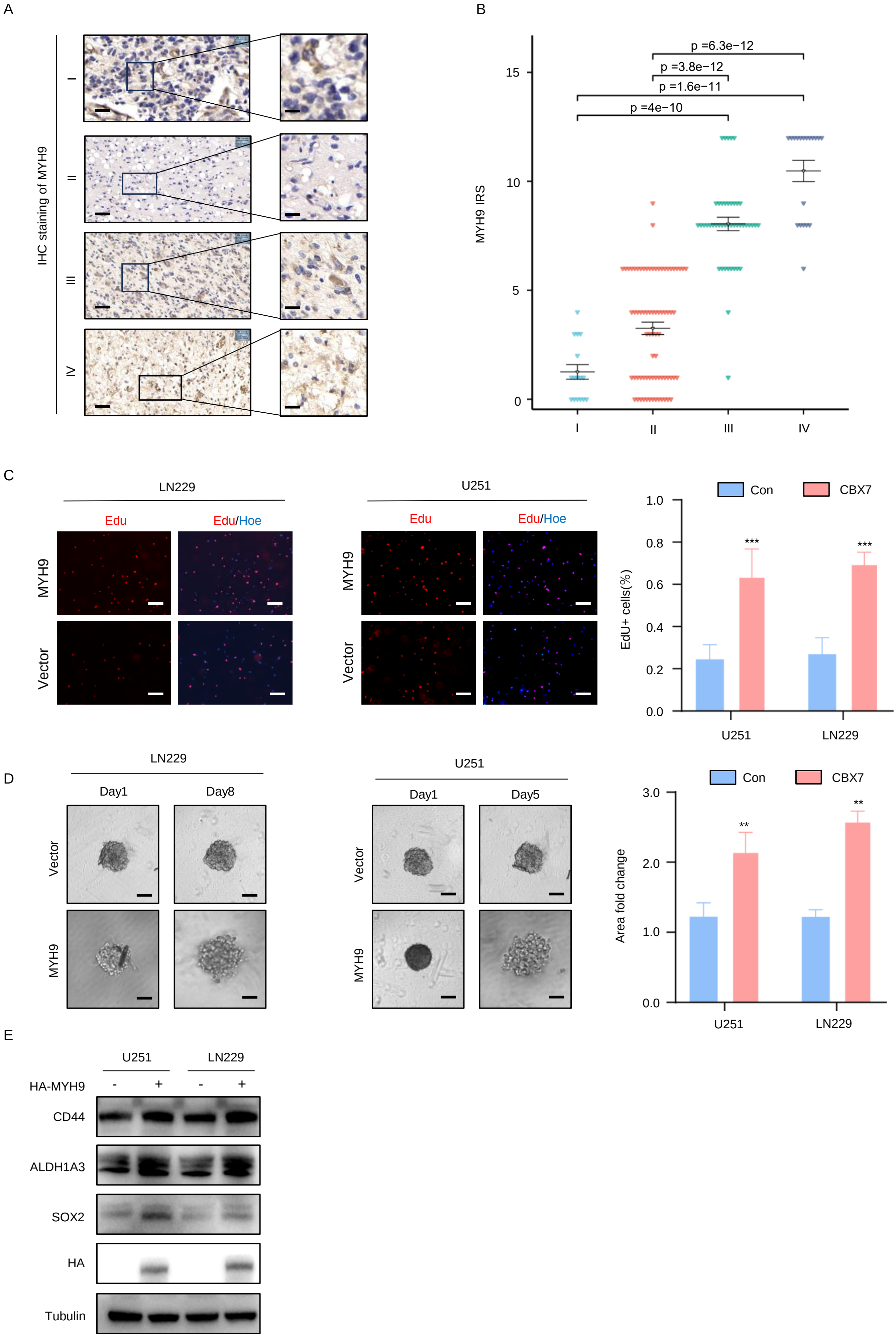

A
B
IHC staining of MYH9
I
II
III
IV
p =6.3e−12
p =3.8e−12
p =1.6e−11
p =4e−10
MYH9 IRS
15
10
5
0
I
II
III
IV
C
LN229
U251
Edu
Edu/Hoe
MYH9
Vector
Con
CBX7
EdU+ cells(%)
1.0
0.8
0.6
0.4
0.2
0.0
***
***
U251
LN229
D
LN229
U251
Day1
Day8
Day1
Day5
Vector
MYH9
Con
CBX7
Area fold change
3.0
2.0
1.0
0.0
**
**
U251
LN229
E
U251
LN229
HA-MYH9
-
+
-
+
CD44
ALDH1A3
SOX2
HA
Tubulin

Supplementary Figures

Figure S1. Knockdown CBX7 promotes glioblastoma cell stemness

A. Relative protein levels of CBX7 in glioblastoma cell lines and the Human embryonic kidney cell line 293T.

B. Western blot analysis of ectopic expression and knockdown of CBX7 in stable cell lines. Tubulin served as a loading control.

C. RT-qPCR analysis revealed upregulation of ectopic CBX7 and downregulation following knockdown of CBX7 in stable cell lines (***$p < 0.001$, Student's t-test).

D. Cell growth assay of indicated stable cell lines using CCK-8. (mean ± SD, n = 4, **$p < 0.01$, Student's t-test).

E. Colony formation in control or CBX7 expressing cells following knockdown (left) show a significant increase in the number of colonies following knockdown of CBX7 (right; n = 4, **$p < 0.01$, ***$p < 0.001$, Student's t-test).

F and G. Transwell migration (**F**) and invasion (**G**) assays of the indicated cells (left panel in each figure) show statistically significant increases in both migration and invasion following knockdown of CBX7 (right panel in each image; n = 4, **$p < 0.01$, Student's t-test)

H. 3D tumor spheroid growth of different glioblastoma cell lines. Scale bars = 200 μm.

I. 3D-tumor spheroid growth (left) and quantitative analysis (right) demonstrate that knockdown of CBX7 resulted in significant increases of spheroid growth in T98G and A172 cells (n = 8, ***$p < 0.001$, Student's t-test). Scale bars, 200 μm.

J. Representative western blot analysis of the stem cell markers CD44 and ALDHIA3 in CBX7 or control vector transfected T98G and A172 cells demonstrate that knockdown of CBX7 results in an increase in the stem cell markers.

Figure S2. CBX7 regulates MYH9 through the PRC1 complex

A and B. Correlation analysis of CBX7 and MYH9 at the mRNA level in the Gravendeel-GBM and CGGA-GBM datasets reveals that CBX7 exerts no effect on MYH9 mRNA levels (Spearman's correlation analysis, p value and correlation coefficient R value are as indicated).

C and D. The mRNA levels of MYH9 in U251 cells transfected with the CBX7 overexpression or empty plasmid (n = 8, Student's t-test, **p < 0.01, ***p < 0.001, ns, not significant).

E. Representative images of co-transfection experiments with the indicated plasmids in 293T cells. Ubiquitinated MYH9 was detected via immunoprecipitation with an anti-Myc antibody (MG132: 10 μM).

F. Co-transfection of 293T cells with the indicated plasmids revealed ubiquitination of MYH9 via immunoprecipitation with an anti-Myc antibody (MG132: 10 Mm, RB3: 8 Mm).

Figure S3. MYH9 activates and CBX7 inhibits the NF-κB signaling pathway via MYH9

A. Luciferase assay of NF-κB transcriptional activity in 293T cells. Co-transfection of 293T cells with a luciferase reporter gene plasmid for NF-κB and an MYH9 overexpression plasmid demonstrated that whilst MYH9 increased NF-κB signalling, the addition of blebbistatin inhibited the regulatory effect of MYH9 on NF-κB signalling. Blebbistatin (8 ng/ml) was added 24 h prior to the assay (n = 4, **p < 0.01, ns, not significant, Student's t-test).

B and C. Representative western blot analysis of NF-κB signaling pathway markers P65, IκBα and p-P65, p-P65 in T98G and A172 cells transfected with the indicated plasmids.

D. Luciferase assay of NF-κB transcriptional activity was detected in indicated cells after transfection of the indicated plasmid (n = 4, Student's t-test, **p < 0.01).

E. Representative western blot analysis of NF-κB signaling pathway markers P65, IκBα and p-P65, p-P65 in T98G and A172 cells transfected with the indicated plasmids.

Figure S4. MYH9 exerts a biological function of pro-cancer in glioblastoma

A. Immunohistochemical (IHC) analysis of MYH9 protein expression in a tissue array containing primary glioblastoma tumors of different pathologic grades. Scale bars, 20 μm

B. Analysis of MYH9 protein expression in glioblastoma tumors of different pathological grades reveals that increases in MYH9 protein is associated with pathological grade.

C. EdU-labeling assay detecting DNA replication in LN229 and U251 cells. Overexpression of MYH9 in these two cell lines resulted in a significant proliferation of cells (n = 8, ***p < 0.001, Students t-test). Data is expressed as the percentage of EdU positive cells (Red) to total cells indicated by Hoechst labeling.

D. Overexpression of MYH9 in LN229 (left panel), and U251 (middle panel) 3D-tumor spheroids resulted in significant growth of spheroid size (right panel) compared to empty vector transfected cells (n = 8, **p < 0.01, Students t-test). Scale bars, 200 μm.

E. Representative western blot analysis of stem cell markers CD44, ALDHIA3 and SOX2 in U251 and LN229 cells transfected with an MYH9 plasmid revealed an increase in stem cell marker expression.
