# Supplementary figures and images for: Chromobox protein homolog 7 suppresses the stem-like phenotype of glioblastoma cells by regulating the myosin heavy chain 9-NF-κB signaling pathway
Source: Cell Death Discov. 2025 Feb 23;11:74. doi: 10.1038/s41420-025-02362-7 (PMC11847914; doi:10.1038/s41420-025-02362-7)

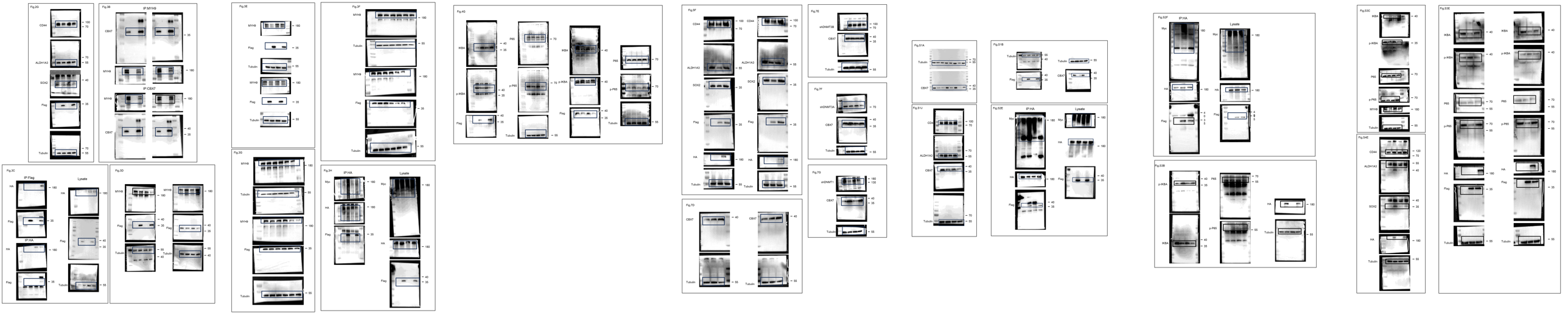

Supplement: Supplementary file 6 — Original western blots [file 41420_2025_2362_MOESM6_ESM.png]
